# Sustainable nanomaterials: the role of Cyrene in optimising carbon nanotubes dispersion and filtration efficiency

**DOI:** 10.3389/fchem.2024.1498279

**Published:** 2024-12-19

**Authors:** Roxana A. Milescu, C. Rob McElroy, Edward J. Taylor, Peter Eaton, Paul M. Williams, Richard Phillips, Thomas J. Farmer, James H. Clark

**Affiliations:** ^1^ Circa Renewable Chemistry Institute, Department of Chemistry, University of York, York, United Kingdom; ^2^ Department of Chemistry, School of Natural Science, University of Lincoln, Lincoln, United Kingdom; ^3^ Department of Biological and Life Sciences, School of Natural Science, University of Lincoln, Lincoln, United Kingdom; ^4^ The Bridge, University of Lincoln, Lincoln, United Kingdom; ^5^ Department of Chemical Engineering, Faculty of Science and Engineering, Swansea University Bay Campus, Swansea, United Kingdom; ^6^ Membranology Limited, Swansea, United Kingdom; ^7^ Green Chemistry Centre of Excellence, Department of Chemistry, University of York, York, United Kingdom

**Keywords:** Cyrene, carbon nanotubes, nanofluid, nanocomposite, buckypaper, filtration membrane, sustainability, renewable chemistry

## Abstract

This study focuses on the fabrication and characterisation of single-walled carbon nanotube (SWCNT) buckypapers and polyethersulfone (PES) flat-sheet membranes using Cyrene, aiming toevaluate its efficacy as a green solvent for these applications. Pristine SWCNTs were dispersed inCyrene without surfactants and compared to N-Methyl-2-pyrrolidone (NMP) dispersions. Buckypapers were fabricated from these dispersions and characterised using Scanning ElectronMicroscopy (SEM), Atomic Force Microscopy (AFM), and infrared spectroscopy. Their performancewas tested in wastewater and oil-water emulsion filtrations and antimicrobial activity. PESmembranes incorporating SWCNTs were prepared using phase inversion and analysed via SEM,optical microscopy, and contact angle. Membrane properties and water permeability were assessed,and bacterial challenge tests evaluated antimicrobial activity. Cyrene enabled the dispersion ofSWCNTs at higher concentrations (0.038 mg mL⁻^1^) compared to NMP (0.013 mg mL⁻^1^). Transmission Electron Microscopy (TEM) analysis revealed that Cyrene effectively debundles SWCNTs, yielding better dispersion. Buckypapers fabricated with Cyrene demonstrated dense, uniform networks with enhanced surface smoothness and promising filtration performance for wastewater treatment and oil-water separation. PES membranes made with Cyrene exhibited well-organised finger-like structures, interconnected pores, superior porosity, and higher water permeability than NMP-based membranes. Incorporating SWCNTs further improved membrane performance. However, bacterial challenge tests indicated no significant antimicrobial activity. The findings highlight Cyrene’s potential as a sustainable alternative to traditional solvents, offering improved material properties and filtration performance. Despite these advantages, further studies are necessary to address solvent residuals and long-term safety considerations, ensuring its suitability for broader applications.

## 1 Introduction

**FIGURE 1:**
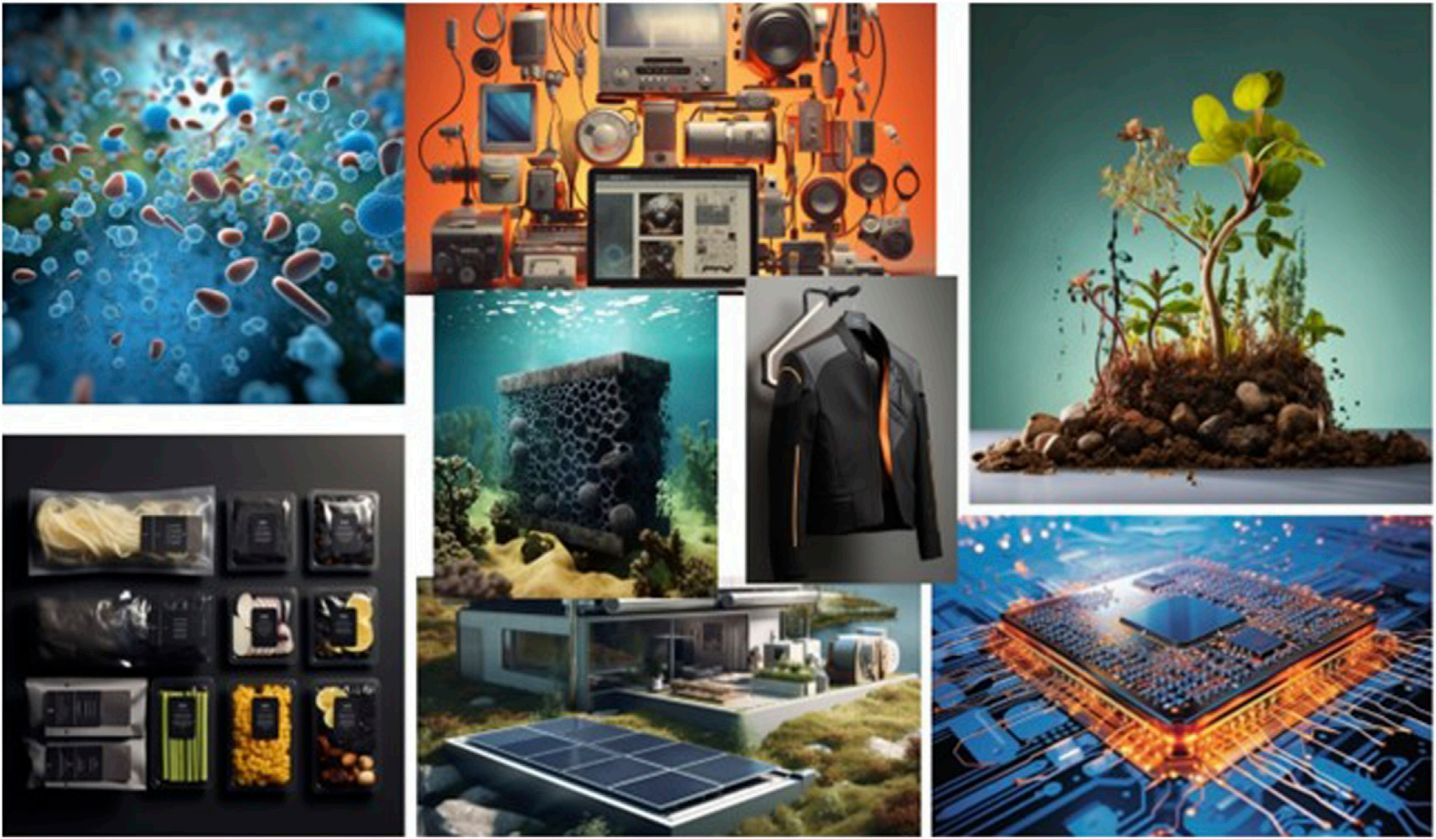
Applications of carbon nanotubes ranging from electronics and energy storage to food packaging, plant growth, smart clothing, and filtration. Photographs realised by the author using AI tool Midjourney.

Nanotechnology has emerged as a groundbreaking field with transformative potential across various scientific disciplines, and its application in food science stands as a testament to its versatility and impact. Nanoparticles have been applied in diverse applications across multiple industries due to their exceptional strength, thermal and electrical conductivity, and antimicrobial properties (Baig, Kammakakam, and Falath, 2021). Carbon nanotubes (CNTs), particularly, have been widely used since their discovery in 1991, in advanced materials and composites, electronics and nanotechnology, energy storage, conductive films and coatings, medical applications, mechanical and aerospace applications, thermal management, filtration, sensors and medical devices and catalysis (Figure 1) (Saeed, 2013; Choi, Yun, and Hwang, 2023; Majeed, 2023).

CNTs are incorporated into composites to improve strength, stiffness, thermal stability and conductivity. These properties have been particularly useful in the development of lightweight and high-performance materials, especially in aerospace (Siochi, 2017; Bellucci et al., 2004; Rawal, Brantley, and Karabudak, 2013). The exceptional electrical conductivity of carbon nanotubes is pivotal in their widespread application in nanoelectronics and nanotechnology. Their structure creates a delocalised *π*-electron system along the tube, allowing electrons to move freely across the lattice (Conley and Karttunen, 2022). They need to be high-quality CNTs, otherwise, defects, such as vacancies or irregularities in the carbon lattice, can impede electron movement. CNTs’ electrical properties position them as promising candidates for next-generation electronic devices, finding applications in transistors, interconnects, sensors, conductive films, and more (Endoh, 2015; Gruner, 2006; Jensen et al., 2004; Sen et al., 2020). Leveraging their high surface area and conductivity, CNTs are also being explored for energy storage applications, aiming to enhance the performance of batteries and supercapacitors in terms of storage capacity and charge/discharge rates (Zhou, Sun, and Su, 2005; Chen et al., 2012; Y.J; Xu et al., 2010). Incorporating carbon nanotubes into food science could revolutionise food safety, preservation, and nutrition by enabling antimicrobial packaging, freshness-extending barriers, and efficient nutrient delivery systems. (Vithanage et al., 2017; R.Q; Liu and Lal, 2015). CNTs can adsorb pollutants and contaminants from water, making them useful for water purification applications (Tiraferri, Vecitis, and Elimelech, 2011; Q.B; Xu et al., 2022). Carbon nanotubes have become the premier candidate in polymeric membrane modification owing to their outstanding mechanical and thermal properties, unprecedented hollow structure, and large specific surface area (Guo et al., 2019). Membranes using carbon nanotubes have shown antifouling and antimicrobial properties (Maksimova, 2019; Tiraferri, Vecitis, and Elimelech, 2011). The antibacterial action of CNTs is believed to involve a combination of physical and chemical mechanisms and is influenced by diameter, lengths, residual catalyst, the presence of functional groups on their surface, and their electronic structure (Saleemi et al., 2022; Jackson et al., 2013). Single-walled carbon nanotubes (SWCNTs) are known to exhibit higher antibacterial activity than multi-walled carbon nanotubes (MWCNTs) due to their smaller size (diameter) that facilitates the partitioning and partial penetration into the cell wall (Kang et al., 2008; Smith and Rodrigues, 2015). CNT-based filtration membranes offer high efficiency and unique properties, yet they face several challenges: 1) aggregation and dispersion (CNTs aggregates can create an uneven distribution and block filtration pathways, affecting the permeability and fouling resistance of the membrane); 2) structural integrity and stability (CNTs may detach or shift during filtration, leading to membrane degradation and release of nanotubes into the filtered water); 3) membrane fouling (biofouling and scaling remain challenges that can reduce membrane efficiency over time); 4) high production costs; 5) health and environmental concern (due to the potential release of CNTs into the environment or drinking water raises safety concerns); 6) scalability and reproducibility (producing CNT membranes with consistent quality and performance on a large scale is challenging and even minor variations in synthesis can affect the membrane performance). Addressing these issues will be crucial for advancing CNT-based filtration technologies to widespread, safe, and economically viable applications.

The simpler version of filtration membranes are the buckypapers, which are flexible thin sheets or films of entangled or aligned carbon nanotubes (Rani et al., 2023). They are normally produced by chemical vapour deposition, dip-coating, vacuum or pressure-based filtration of dispersions of carbon nanotubes in solvents and surfactants (Mustafa et al., 2017; Park et al., 2009; Wang et al., 2008; Zhang et al., 2005; Wu et al., 2004). Despite the promising applications, there is widespread apprehension regarding the toxicity and cytotoxicity associated with carbon nanotubes. Respiratory exposure to carbon nanotubes was found to cause lung toxicity, leading to lung injury and formation of tumor (Muller, Huaux, and Lison, 2006). Inhalation of nanotubes leads to lung carcinogenicity in some cases, and organ damage in the liver, kidney, brain, spleen and more (Kasai et al., 2016; Francis and Devasena, 2018). Moreover, they could also induce damage to the genetic material and death in some species from aquatic life, bacteria and some plants (Du et al., 2013; Mohanta et al., 2019; X.Y; Liu et al., 2009; Mitchell et al., 2007). The toxicity of CNT to bacteria is currently being exploited in a range of applications including filtrations, toxicity towards eukaryotic organisms remains a crucial factor to be considered when employing them at scale and in applications involving human contact.

Achieving a well-dispersed solution of carbon nanotubes is essential, as it optimises their surface area, uniform distribution, and functional properties, all of which are critical for high-performance applications. When carbon nanotubes aggregate into bundles, key properties, such as conductivity, mechanical strength, and chemical reactivity, are significantly restricted, thereby diminishing their overall effectiveness and application potential. This aggregation occurs due to van der Waals and π-π interactions, resulting in the formation of ropes or bundles, which can further entangle in aqueous solutions. Their dispersion in media involves various interactions, and SWCNTs have a more hydrophobic surface compared to MWCNTs due to their perfect sp^2^ backbones, affecting their processability. To achieve efficient debundling and stabilisation of carbon nanotubes, their surface is typically modified through covalent methods, such as oxidation, halogenation, radical addition, or through non-covalent approaches like adsorption of carbohydrates, proteins, surfactants, or polymers (Naqvi et al., 2020; Yadav, Gupta, and Sharma, 2022). Although covalent surface functionalisation improves dispersibility in pure solvents, it can also cause disruptions to the graphitic structure of the walls, which may require further correction. These defects can affect the electrical and mechanical properties of the functionalised carbon nanotubes. The stability of non-covalently functionalised carbon nanotube suspensions depends on factors such as the type and concentration of dispersants, the nanotube length, and the surfactant properties. Moreover, surfactants can pose challenges as they are often toxic and difficult to remove from the fluid. (Vahid et al., 2018; Kharlamova, Paukov, and Burdanova, 2022).

**FIGURE 2:**
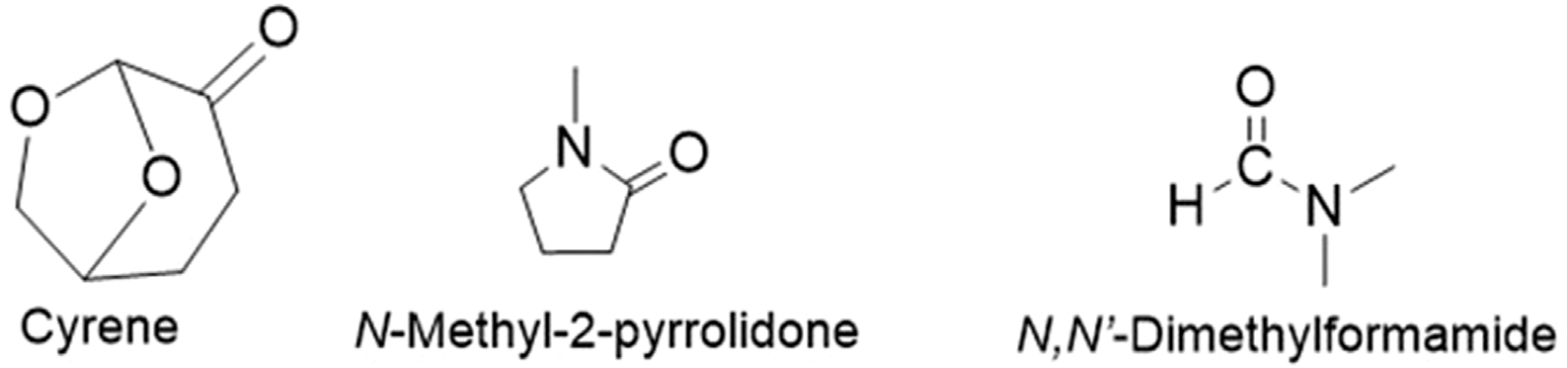
Chemical structures of Cyrene, *N-*methyl-2-pyrrolidone, and *N, N’*-dimethylformamide.

CNTs are generally dispersed in amide solvents such as DMF and NMP, which come with associated health and environmental problems (Ausman et al., 2000; Giordani et al., 2006; J; Liu et al., 1999). In recent years, the quest for sustainable and environmentally friendly alternatives in the field of chemistry has gained substantial momentum. Amid this drive, the polar aprotic solvent Cyrene has emerged as a notable contender, promising to revolutionise the landscape of solvent-based processes (Camp, 2018; Sherwood et al., 2014). The chemical structure of Cyrene is seen in Figure 2, along with those of NMP and DMF.

Derived from renewable cellulose sources, Cyrene embodies a unique blend of green chemistry principles and versatile solvent properties, making it an attractive candidate for a wide range of applications (Milescu et al., 2019; Milescu et al., 2020; Meng et al., 2020; Sullivan et al., 2022; De Bruyn et al., 2019; Warne et al., 2023). Cyrene has been previously reported in a non-covalent dispersion of MWCNTs with the aid of electron-deficient acceptors which had the role of interrupting the inter -CNTs *π*-*π* interactions via a donor-acceptor interaction mechanism, permitting the debundling and dispersion of individual MWCNTs and inhibiting their re-agglomeration (Gharib et al., 2018). Moreover, Cyrene was used as a dispersing medium for MWCNT-based supercapacitor electrodes, giving stable suspensions of relatively high-concentration nanotubes without the need for a surfactant (Poon and Zhitomirsky, 2020). In recent research, the stability of a Cyrene nanofluid containing SWCNTs was found to be due to the weak van der Waals interactions between the solvent and the SWCNTs through X…π interactions (Das et al., 2023). Cyrene has been effectively employed as an exfoliating agent to fabricate monolayer and few-layered structures of 2D materials, including graphene and transition metal dichalcogenides (TMDs) (Salavagione et al., 2017; Adam et al., 2023). Its high solvency power and low toxicity make Cyrene a sustainable alternative to traditional solvents like NMP and DMF. Cyrene facilitates the liquid-phase exfoliation process, ensuring high-quality dispersions without requiring surfactants. This approach yields stable colloidal suspensions of graphene and TMDs with potential applications in electronics, energy storage, and environmentally conscious material fabrication.

In this study, pristine SWCNTs were dispersed in the environmentally friendly solvent Cyrene, as well as NMP, to fabricate buckypapers for the first time without the use of surfactants. These concentrated nanofluids were subsequently employed to produce flat-sheet membranes. The resulting buckypapers and membranes were rigorously evaluated for their performance in filtration applications.

## 2 Experimentation

### 2.1 Materials

Single-walled carbon nanotubes (SWCNTs) used in this project were kindly offered by OCSiAl and had ˂2 nm diameter and >5 µm length. The nanotubes concentration is ≥ 80% and impurities account for ≤15%. Cyrene was kindly supplied by Circa Group. NMP was purchased from Acros Organics. Ultrason E3020 P Polyethersulfone (PES) of 55,000 Da and polyvinylpirrolidone Luvitek K-90 (PVP) Pulver of 1,500,000 Da were obtained from INGE.BASF, Germany. Both polymers were solvent exchanged in ethanol and ethyl acetate respectively and dried in a vacuum oven overnight before use. Strains, materials and reagents for the antimicrobial activity assessment (*Escherichia coli* XL10-Gold and *Bacillus subtilis* 168, DSM 402) were sourced from Agilent and Leibniz Institute DSMZ respectively. Whatman Filter Papers Grade 1 and phosphate-buffered saline (PBS) Tablets were sourced from Thermo Fisher Scientific. Miller Luria Broth and Agar were purchased from Melford and quaternary ammonium compound-based disinfectant (Q SHIELD Surface Care Spray was sourced from Q Biotechnologies.

### 2.2 Single-walled carbon nanotubes dispersion

Dispersions of 0.1% pristine SWCNTs in Cyrene and NMP (w/v) were prepared using a bath sonicator (GT SONIC-D3 with an ultrasonic power of 100 W) for 1 hour in water maintained at a temperature below 25°C (Supplementary Figure S1). 15 mL centrifuge tubes containing the desired solvent and SWCNTs were placed in a beaker, which in turn was placed into an ultrasonic bath and sonicated for an hour. After sonication, the samples were centrifuged for 10 min at 2,500 g, at room temperature using a Biofuge Primo centrifuge with an angle of 90° and 17 cm rotor. The upper 80% of the supernatant was then carefully decanted, separating the big bundles of SWCNT ropes and impurities from the commercial nanotubes. The supernatant was then collected and filtrated through 0.22 µm pore size nylon membranes with a diameter of 13 mm (pastel green P/N: FIL-S-NY-022-13-100 from Chromatography Direct). The nanotubes deposited onto the membrane were washed three times with 10 mL ethanol to remove any residual solvent and left to dry in the air for several days. To calculate the concentration of SWCNTs dispersed, the SWCNTs retained from the filtration membrane were recovered, dried, and weighed and the actual concentration registered.

### 2.3 Buckypaper production by pressurised filtration

The synthesis of buckypaper was accomplished through a straightforward filtration process. A dispersion of 1.25% w/v SWCNTs (plural form for single-walled carbon nanotubes) in Cyrene was achieved by employing the identical ultrasonic bath described in Section 2.2 for a duration of 1 hour. Following dispersion, the sample underwent centrifugation (10 min at 2,500 g, at room temperature, using a Biofuge Primo centrifuge with an angle of 90° and 17 cm rotor.). The resulting supernatant was then collected and filtrated through a 0.22 µm pore size nylon membrane with a diameter of 13 mm. The retained nanotubes on the filter were subsequently subjected to three ethanol washes (10 mL each) and left to air-dry at room temperature.

### 2.4 Filtration tests using the Cyrene-based buckypaper

The surfactant-free Cyrene-based buckypaper, deposited on the nylon membrane, was employed to filter household wastewater and effect the separation of water and cooking oil from an emulsion. A sample of household wastewater and a 1:1 water:oil emulsion were subjected to filtration using a syringe equipped with the buckypaper, deposited atop a 0.45 μm nylon membrane.

### 2.5 Polymeric flat-sheet filtration membranes preparation

The CNT-based flat-sheet PES membranes have been manufactured via non-solvent induced phase separation process (NIPS), using water as a coagulant. In this project, four membranes have been prepared, and their casting solution composition can be seen in Table 1.

**TABLE 1:** Casting solution composition (wt%) of PES membranes produced with polyethersulphone (PES) as main polymer in NMP or Cyrene and with/without additives (SWCNTs and PVP).

Membrane type	Polymer and additive (wt%)			Solvent (wt%)	
	PES	PVP	SWCNT	Cyrene	NMP
PES/C0	15	0	0	85	0
PES/N0	15	0	0	0	85
PES/CNT/C0	15	0	0.1	84.9	0
PES/CNT/C3	12	3	0.1	84.9	0
PES/CNT/C5	10	5	0.1	84.9	0
PES/CNT/N0	15	0	0.1	0	84.9

Single-walled carbon nanotubes are simply labelled as “CNT.” “PES/CNT/C0” is the membrane produced with Cyrene and single-walled carbon nanotubes but no additive. “PES/CNT/N0” is manufactured using NMP, and single-walled carbon nanotubes but no additive. PES/CNT/C3 and PES/CNT/C5 mean a membrane produced with 3% and 5% PVP, respectively, using Cyrene as solvent and containing single-walled carbon nanotubes. All “CNT’’ membranes have been produced using 0.1% pristine single-walled carbon nanotubes.

The SWCNTs were dispersed first in the solvent by sonicating for 1 hour. This step was skipped for the pristine membranes. This solution was used to dissolve PES by dissolving 10–15 wt% of PES pellets at a temperature of 70°C for 4 h. Then concentrations of 3% PVP or 5% PVP were added under continuous stirring. The casting solution was degassed and then placed on a glass plate and a film of thickness of 150 µm was obtained using a manual casting knife. The casting film was submerged in a coagulation bath containing deionised water at RT. Membranes were then washed three times in distilled water for 10 min while under sonication in order to wash out the residual solvent. The fabricated membranes were then stored in deionised water until further use. To characterise the prepared membranes, they were dried in a vacuum oven at 80°C overnight.

### 2.6 Scanning Electron Microscopy (SEM) analysis

The PES flat sheet membranes were dried and frozen in liquid nitrogen followed by Au/Pd coating. Cross-sectional SEM images of the coated membranes were recorded using a JEOL JSM-6490LV Scanning Electron Microscope. SEM images of the surface of one membrane and of the buckypapers were measured using a Thermoscientific Scios 2 scanning electron microscope, EDX analysis was performed on this instrument using a Bruker XFlash 6.60 EDX detector. All images shown here are secondary electron images.

### 2.7 Transmission electron microscopy (TEM) analysis

200 mesh formvar/carbon copper grids were pre-treated by glow discharge in Polaron E6000 vacuum coating unit to make them hydrophilic. 5 μL of each sample were loaded onto these pre-treated grids and left to dry for 48 h in a fume hood before analysis. The grids were analysed on FEI Tecnai 12 BioTwin G2 Transmission Electron Microscope operating at 120 kV. The images were collected on a SIS CCD camera at magnifications of 6.8 k and 98 k.

### 2.8 Optical microscopy

Optical microscopy images were recorded using a Leica S6D Microscope with 6.3×-40× magnification and flat image field after placing a droplet of sample (wastewater or emulsion solution before and after filtration) onto a glass slide. The working distance is 110 mm, from microscope to specimen, providing space for manipulation.

### 2.9 Atomic force microscopy (AFM)

For AFM, uncoated samples were used. The samples were scanning using an AFMWorkshop HR-AFM instrument in vibrating mode. Vibrating mode cantilevers with resonant frequency about 200 kHz were used. Images were collected in 6 × 6 µm areas of each sample, and all images shown here are height images.

### 2.10 Water flux tests

**FIGURE 3:**
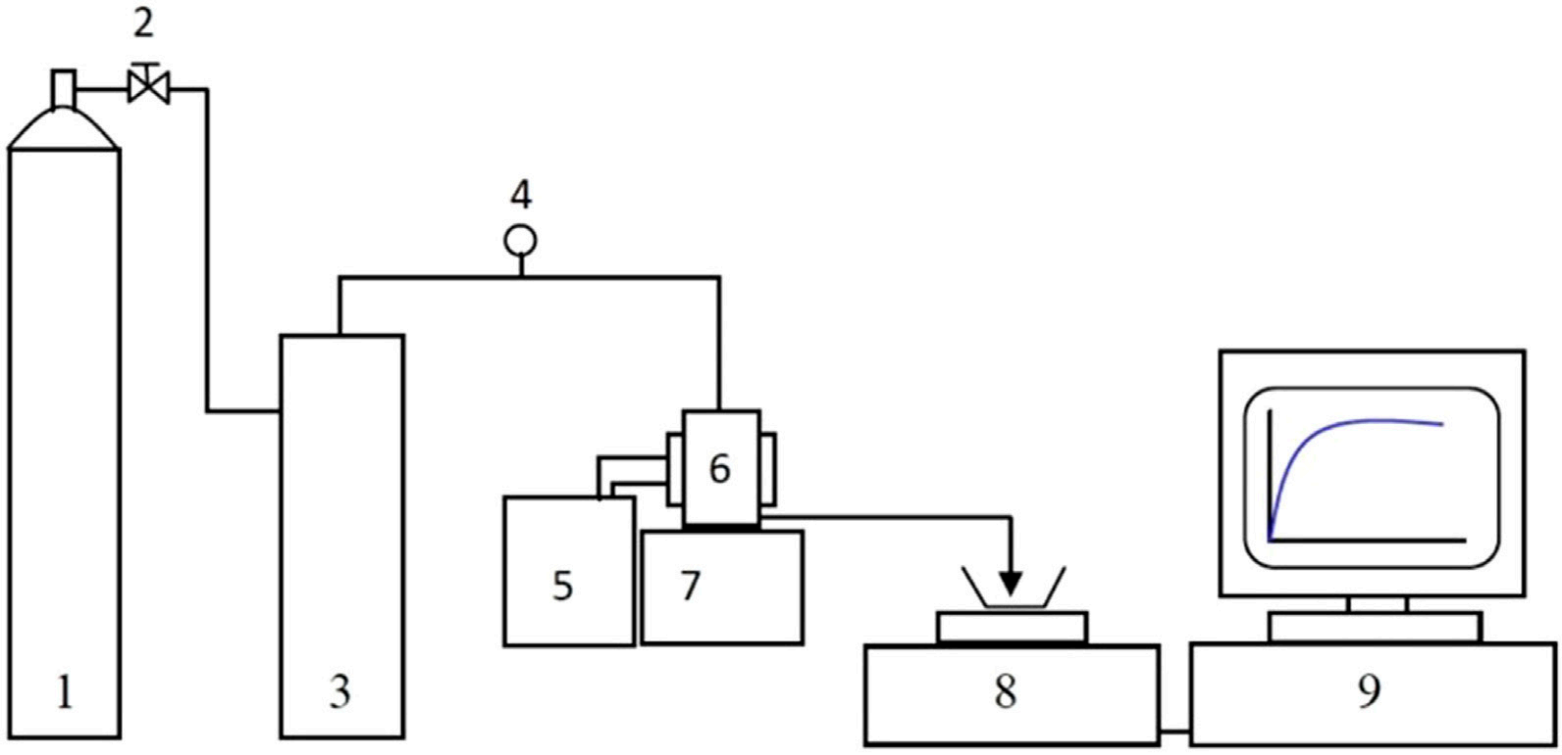
A schematic diagram of the frontal filtration equipment: nitrogen cylinder (1), valve (2), feed reservoir (3), pressure sensor (4), water bath (5), membrane cell (6), magnetic stirrer (7), electronic balance (8), PC (9).

Dead-end membrane filtration measurements were carried out using a Sterlitech HP4750 membrane filtration cell (max volume 300 mL) connected to a nitrogen gas supply. The cell can hold a membrane disk of ∼49 mm in diameter and the active membrane area 14.6 cm^2^. The membrane holder and cell body are made of 316L stainless steel. The stirrer was removed for the pure water flux tests. The arrangement of the filtration and flux measurement equipment is seen in Figure 3.

The filtration cell was pressurized via nitrogen gas (oxygen free) out of a nitrogen cylinder, which was controlled by the reducing valve at the gauge of the cylinder. The applied pressure was monitored by an in-line pressure sensor with an overall measurement error of <1.5%. The cell was not stirred. In this case, rates of filtration *J*_*w*_ (L m^−2^ h^−1^ (LMH)) have been determined via recording the weight of water obtained in a given time interval and then using the Equation 1:

$$J_{w}=\frac{\left(t+∆t\right)-m\left(t\right)\;}{\rho \;Am\;∆t}$$ (1)

where *m* is the mass at a given time, *ρ* is the density of water, *A*_*m*_ is the membrane area and *t* is the time (all in appropriate units to get LMH).

### 2.11 UV-Vis measurements

A UV–Vis spectrophotometer (Shimadzu UV-1800) was used to measure the spectra of wastewater and oil-water emulsion samples before and after filtration by Cyrene-based buckypaper, across a wavelength range of 200–800 nm.

### 2.12 Fourier-transform infrared spectroscopy (FTIR)

The functional groups present in samples were investigated using PerkinElmer Spectrum 400 ATR-FTIR Spectrometer with transmittance peaks in 4,000–500 cm^−1^ region, with rapid scanning (4 scans) and resolution 4 cm^−1^ at room temperature. The obtained data was analysed using the OriginPro 2024 software.

### 2.13 Contact angle measurement

As a measure of membranes’ hydrophilicity, the water contact angle was measured via the sessile drop method using a Theta Lite optical tensiometer at a room temperature of 20°C. A range of 1–2.5 µL droplet sizes of water were placed on the membrane surface and the images were recorded using the automated OneAttension software. The contact angles were measured at a minimum of three random locations and the mean values reported to minimize experimental error.

### 2.14 Anti-bacterial activity

Membrane samples were prepared by cutting 1.3 × 2 cm (2.6 cm^2^) sections. Nylon filter bucky paper was used as is. Samples were autoclaved at 121°C for 20 min to sterilise. 10 mL overnight cultures of *E coli* XL10 Gold and *B. subtilis* 168 were set up in Luria Broth (LB) media and cultured at 37°C shaken at 180 rmm. They were then diluted to an OD of 0.1 (595 nm) in LB media. Using aseptic technique, the membranes were dipped into the diluted culture, and any residual drops were shaken off. The membranes were then placed individually on the inside of a separate sterile 50 ML falcon tube, and spun to remove excess liquid from the membrane, (500 rpm for 1 min). The samples were then incubated for 6 h at 37°C. Membranes were aseptically removed and added to 9.9 mL of sterile phosphate-buffered saline (PBS) and vortexed for 30 s. Serial dilutions were made and plated out on LB agar. The plates were incubated overnight at 37°C and colonies were counted. This was conducted in triplicate for each membrane type. A Whatman NO1 filter paper and nylon filter were included as negative controls and Whatman NO1 filter paper soaked in Quaternary ammonium compound-based disinfectant and then dried, served as a positive control.

## 3 Results and discussion

### 3.1 Single-walled carbon nanotubes dispersion in Cyrene and NMP and the stability of concentrated nanofluids

**FIGURE 4:**
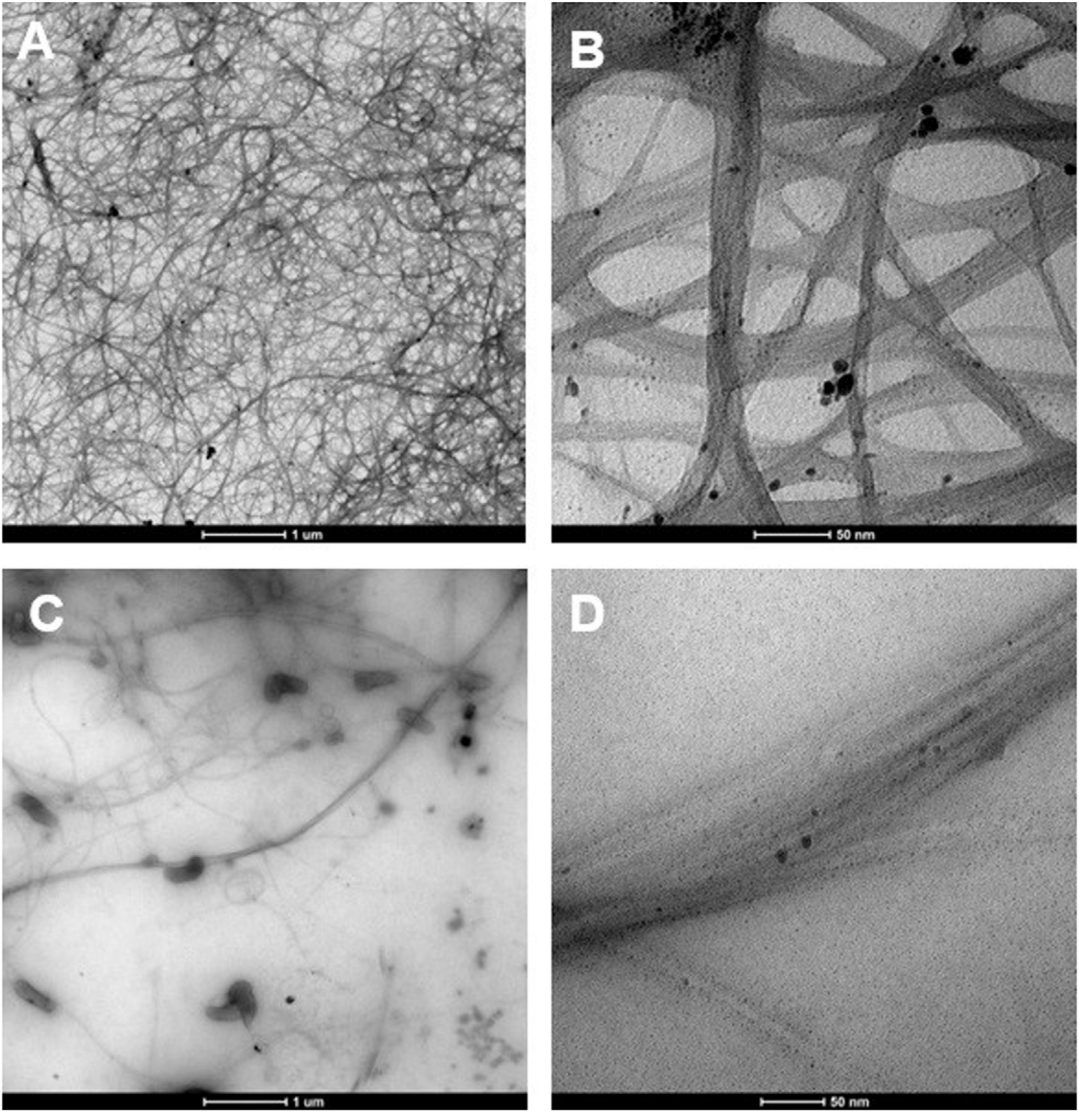
TEM images of 0.1% SWCNT dispersed in neat Cyrene **(A, B)** and NMP **(C, D)** at low **(A, C)** and high resolution **(B, D)** after 1 hour of sonication.

In this study, we investigated the nanofluids of carbon nanotubes (CNTs) dispersed in Cyrene and NMP without the aid of surfactants, focusing on their debundling and stability characteristics. The authors have previously conducted a comprehensive study on the dispersion of single-walled carbon nanotubes (SWCNTs) in Cyrene and NMP, both with and without the inclusion of additives (R. A. Milescu, 2021). Following 1 hour of sonication in Cyrene, the nanotubes were observed across the entire grid surface, indicating effective debundling (unzipping) of aggregates into individual ropes (Figures 4A,B).

Due to the high boiling points of Cyrene (227°C) and NMP (202°C), combined with Cyrene’s significantly higher viscosity (14.5 cP compared to NMP’s 1.6 cP), the solvent tends to remain adhered to the nanotube surfaces. As a result, the walls of the nanotubes cannot be clearly observed. The TEM micrographs reveal the presence of only a few nanotube aggregates, confirming their successful exfoliation in the solvent. In contrast, the NMP-based nanofluid exhibited an uneven dispersion of carbon nanotubes, evident from the gaps on the grid surface (Figures 4C,D). Small bundles of nanotubes can be observed, suggesting an inefficient unzipping to individual ropes. Additionally, both examples showed the presence of small particles of impurities. The Cyrene- and NMP-based nanofluids containing SWCNTs were subjected to stability testing (Supplementary Figure S2). Generally, the stability of particles in a liquid is assessed by monitoring their sedimentation at the fluid’s base due to gravity (Phan and Haes, 2019). A longer settling time suggests higher stability for nanoparticles. In our study, it was noted that the nanoparticles settled within 1 hour in NMP, whereas the process took more time in Cyrene. Supplementary Figure S2A illustrates that, after a day, the Cyrene-based nanofluid exhibited significant SWCNTs agglomeration. The NMP-based nanofluid displayed minimal nanotube presence, evident from its colourless appearance, indicating low debundling and stability (Supplementary Figure S2B). Specifically, the dispersions of pristine SWCNTs in pure NMP proved unstable, with re-agglomeration observed within a short time frame. Surprisingly, even after 1 week, the agglomerates were still suspended in Cyrene, making it a better dispersant than NMP.

Following the stability test, the samples underwent filtration using a 0.22 μm filter, followed by three ethanol washes to eliminate the residual solvent. Subsequently, the filtered material was dried at room temperature overnight. The concentration of dry SWCNTs dispersed in NMP was approximately three times lower than that in Cyrene, measuring 0.013 mg mL^−1^ compared to 0.038 mg mL^−1^ (Supplementary Table S1).

### 3.2 Characterisation and testing of carbon nanotube buckypapers

**FIGURE 5:**
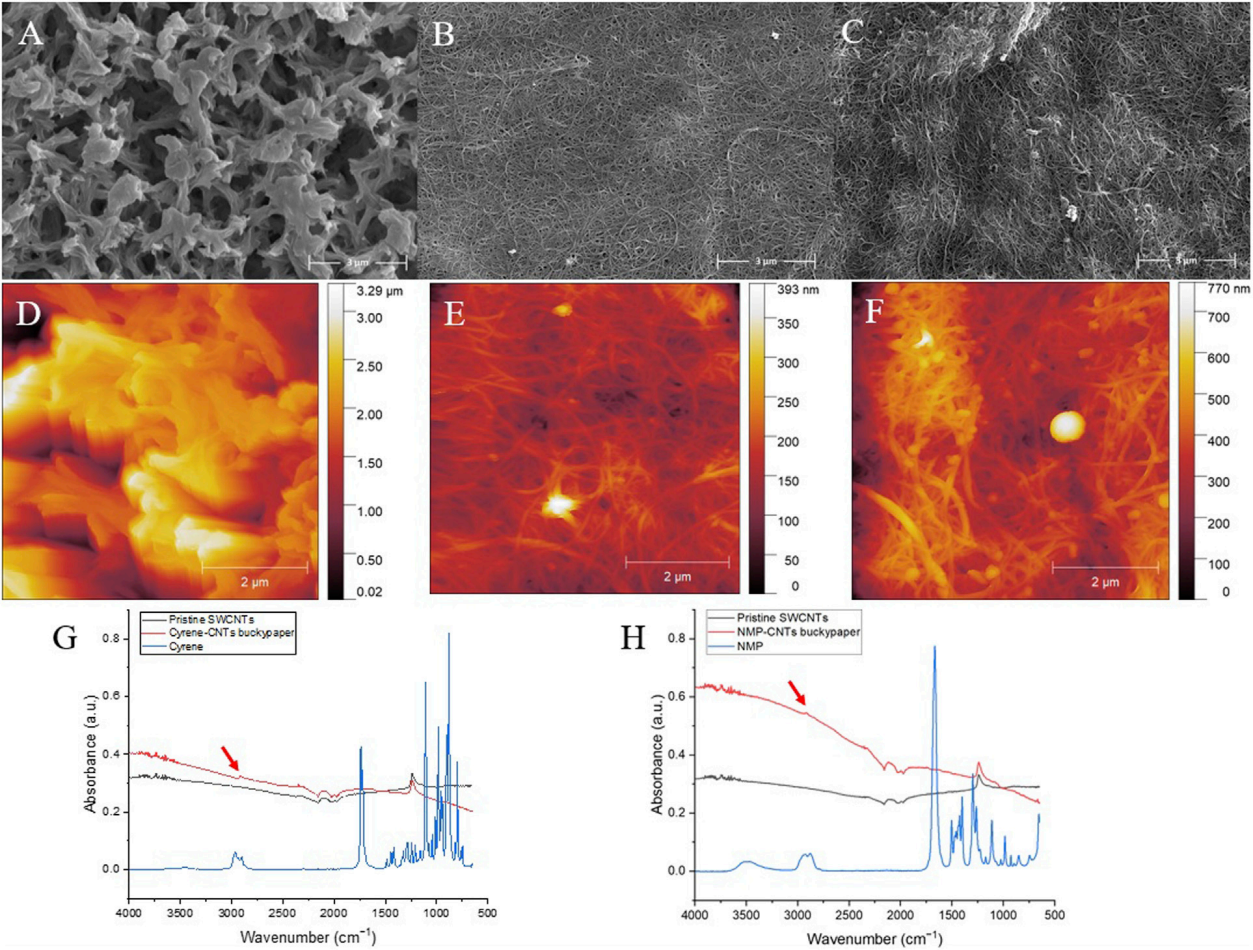
SEM images of the pristine nylon membrane **(A)**, Cyrene-CNTs **(B)** and NMP-CNTs buckypapers **(C)**. AFM height images of pristine nylon membrane **(D)**, Cyrene-CNTs buckypaper **(E)**, and NMP-CNTs buckypaper **(F)**. FTIR spectra of Cyrene-CNTs buckypaper **(G)** and NMP-CNTs buckypaper **(H)** compared to pristine SWCNTs and newt solvents (Cyrene and NMP).

SEM analysis of the buckypapers fabricated using NMP and Cyrene reveals a network of fiber bundles coating the nylon membrane surface (also shown in Figure 5A; Supplementary Figure S3). Cyrene appears to have produced a densely packed layer of SWCNTs (Figure 5B). In contrast, the coating formed using NMP is noticeably thinner, with the underlying membrane surface features still partially visible (Figure 5C).

Additional images in Supplementary Figure S4 also show regions of the NMP buckypaper where the Nylon membrane is clearly visible. These regions were not found on the Cyrene sample (Supplementary Figure S5). Both NMP and Cyrene buckypapers contained a number of impurities visible on their surface (Supplementary Figure S6). Energy-dispersive X-ray spectroscopy (EDX) suggested that these impurities are predominantly iron, in keeping with the data sheets provided by the supplier (Supplementary Figure S7). As such, for certain applications, a preprocessing chemical purification step would be required (Hou, Liu, and Cheng, 2008). The buckypaper exhibits well-defined nanotube ropes and interpores. As no surfactant was employed, no additional additive removal is required, rendering this novel buckypaper type more sustainable. The surface roughness of the buckpapers was compared with that of the nylon membrane (Figure 5D) using atomic force microscopy (AFM). The buckypaper surface was many times less rough than that of the original membrane (see z scales images Figures 5D–F). In keeping with the SEM data, the Cyrene-CNTs buckypaper was also smoother (Figure 5E) than that produced using NMP (Figure 5F). Infrared spectra of both Cyrene- and NMP-based buckypapers revealed residual solvent signals around 3,000 cm⁻^1^, corresponding to O-H and N-H stretching frequencies (Figures 5G,H). The presence of these solvent residues raises concerns regarding the safety of using such filtration materials in applications involving water. This residual solvent could leach out, necessitating further investigation to ensure the safety and suitability of these materials for human-related applications.

**FIGURE 6:**
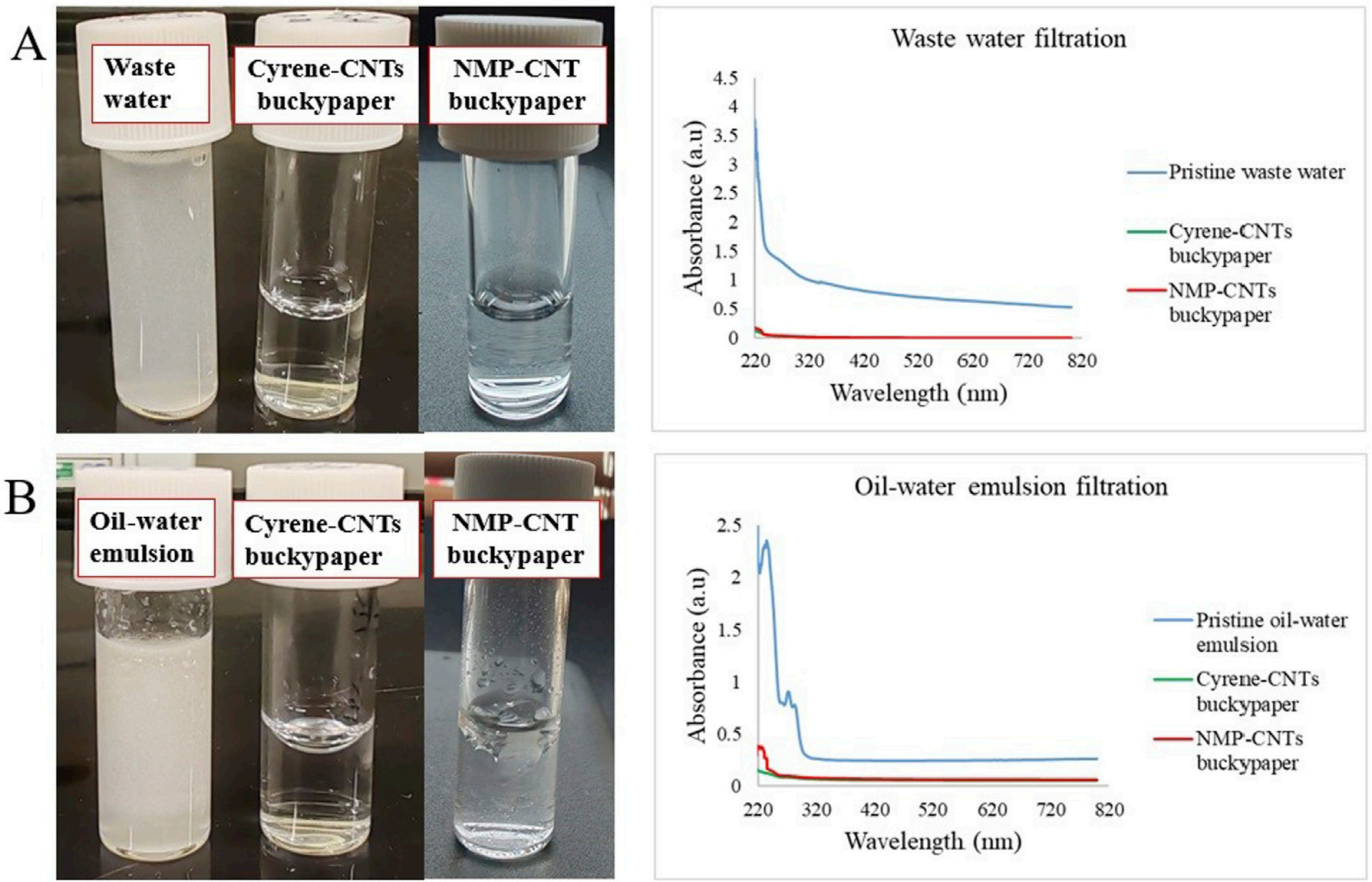
Photographs of the waste-water **(A)** and oil-water emulsion **(B)** and their UV-Vis spectra before and after one-step filtration through Cyrene- and NMP-CNTs buckypapers.

Following the washing and drying process, the Cyrene- and NMP-based buckypapers deposited on a nylon membrane underwent thorough analysis and testing for their filtration capabilities. Two distinct and straightforward filtration applications were chosen for this evaluation: the treatment of waste-water and the separation of a cooking oil-water emulsion. These tests aimed to assess the effectiveness of the Cyrene-based buckypaper in removing contaminants and improving water quality. In the waste-water filtration test, the membrane’s ability to remove suspended solids, and potential pollutants was scrutinised. For the oil-water emulsion separation, the membranes’ performance was evaluated based on its capacity to selectively separate water from oil, thereby achieving a high degree of purity in the filtered water (Figure 6).

As depicted in Figure 6A, the permeate resulting from waste-water filtration revealed clear water samples following a single-step extrusion through a syringe containing the Cyrene- and NMP-based buckypapers. These results align with the UV-Vis spectra of the samples before and after filtration. Although the analysis of complex matrices such as waste-water may not be straightforward, the broad peak area between 220 and 300 nm is significantly reduced after filtration by both buckypapers. In the case of the oil-water emulsion (Figure 6B), a substantial peak reduction is observed after filtration using a Cyrene-based buckypaper. A less effective oil-water separation can be observed in case of NMP-buckypaper with a higher intensity peak observed in the UV-Vis. These results highlight the substantial potential of the Cyrene-based buckypaper, especially in critical scenarios such as accidental oil spillages in marine environments and the purification of other turbid solutions. Moreover, the versatility of this buckypaper extends beyond its application in oil-water separation, opening avenues for its utilisation in various filtration processes across diverse industries. The adaptability and efficacy demonstrated in this study suggest that the Cyrene-based buckypaper holds promise as a versatile solution for addressing environmental challenges and meeting filtration needs in different contexts.

### 3.3 Characterisation and testing of SWCT-based PES flat sheet membranes

Flat-sheet membranes produced with PES, PVP, and Cyrene have been previously reported in the literature by the team (Milescu et al., 2019). It was found that PES membranes produced with Cyrene exhibited higher total porosity than NMP-based membranes and the same pore diameter as typical NMP-based membranes produced with 5% pore-forming additive PVP, highlighting that Cyrene-based membranes do not require pore-forming additives in their composition. In this project, the authors incorporated a low concentration of SWCNTs to enhance the physico-chemical properties of the membranes for applications where high flux and anti-fouling properties are critical. Uniformity was maintained across all carbon nanotube-based membranes, which were produced using a consistent 0.1% mass ratio relative to the solvent. To prevent pore-blocking aggregations of nanotubes, which could negatively affect permeability, we first ensured the dispersion of nanotubes in the solvents before dissolving the polymer in these nanofluids. The flat-sheet membranes containing SWCNTs exhibit distinct visual characteristics when fabricated using Cyrene compared to NMP as solvents (Supplementary Figure S8). In the case of PES/C0 membrane, the surface appears as a wrinkled, dark grey layer. This texture suggests a more compact arrangement of SWCNTs and possibly a unique interaction between the nanotubes and the Cyrene solvent during the fabrication process. On the other hand, PES/CNT/N0 membrane displays a starkly different morphology. The surface features visible “ropes” of nanotubes that stand out through the white polymeric matrix. This transparency highlights a less uniform dispersion of SWCNTs within the polymer matrix. The nanotubes appear to cluster, potentially indicating weaker compatibility or interaction between the SWCNTs and the NMP solvent during processing. This uneven distribution might impact the mechanical, electrical, or permeability properties of the membranes, reflecting differences in the miscibility and solvation dynamics of the two solvents with the polymer and nanotubes.

**FIGURE 7:**
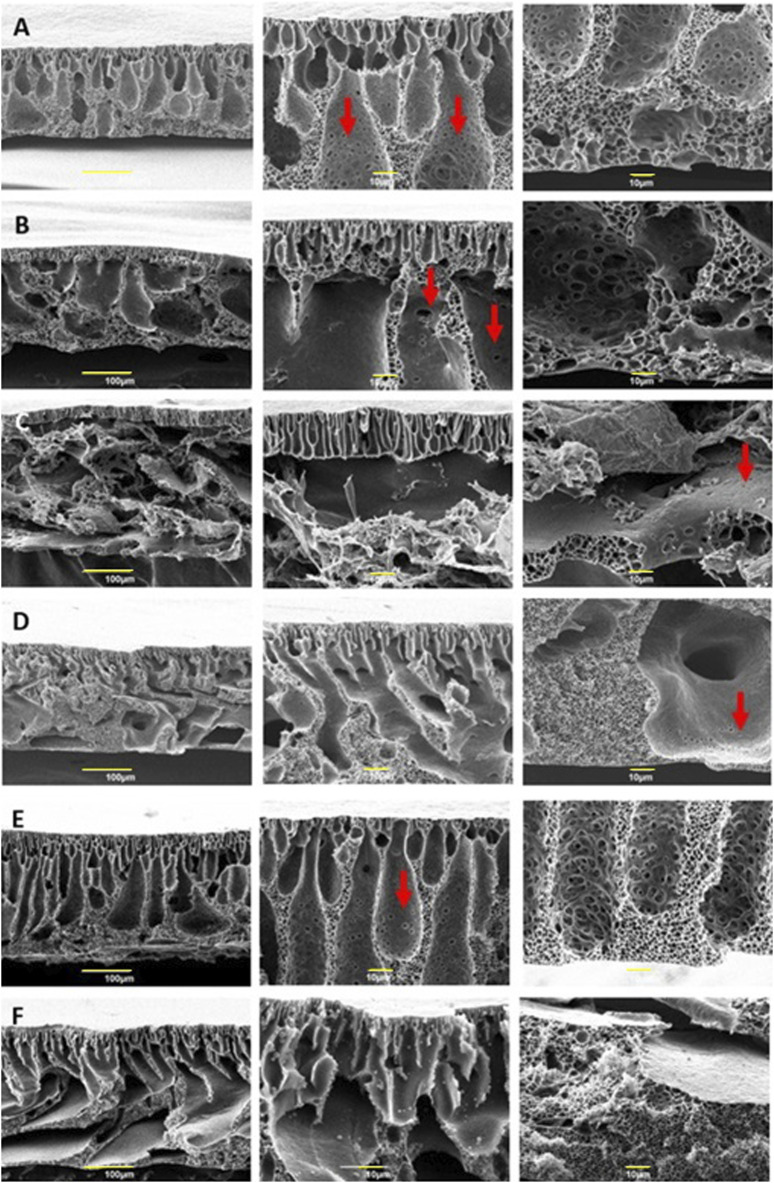
Scanning Electron Microscopy images of cross-section, and details of PES/CNT/C0 **(A)**, PES/CNT/C3 **(B)**, PES/CNT/C5 **(C)**, PES/CNT/N0 **(D)**, PES/C0 **(E)** and PES/N0 **(F)**. All membranes have a thickness of 150 μm.

Pristine PES membranes were produced with Cyrene and NMP for comparison and the cross-section figures of all membranes can be observed in Figure 7.

Commonly referred to as “Loeb-Sourirajan” membranes, this type is characterized by a thick, porous surface and an asymmetric distribution of pore sizes and porosity across the membrane’s thickness. Notably, smaller voids are present near one surface, while larger ones are evident on the opposite surface (Loeb and Sourirajan, 1962). The top layer of the membranes is supported by a sponge-like substructure, resulting from a slow demixing, and macrovoids formed due to instantaneous demixing during phase inversion. Finger-like structures can be observed near the top layer of the membrane, resulting from rapid demixing between the solvent (Cyrene or NMP) and non-solvent (water). The finger-like channels observed in membranes fabricated using Cyrene exhibited a more pronounced vertical orientation and a well-organised structure compared to those produced with NMP. Additionally, membranes prepared from Cyrene showcased a higher density of interconnected pores within the walls of the finger-like channels, consistent with prior findings (Ferreira et al., 2017). The porosity of PES/CNT/C0 (Figure 7A) is different from PES/CNT/N0 (Figure 7D) for the sponge structure; larger pores are observed in the Cyrene-based membrane. This phenomenon of pores forming in Cyrene without the aid of an additive was previously reported (Milescu et al., 2019). The introduction of PVP, commonly employed as a pore-forming additive, resulted in the formation of macrovoids in the Cyrene-based membranes (Figures 7B,C). The incorporation of PVP led to a reduction in sponge-like structures, with the extent of this reduction correlating with the amount of additive introduced into the casting solution. PES/CNT/C3 (Figure 7B) membrane reveals diminutive finger-like structures near the upper surface, a sponge-like configuration adjacent to the lower side (where the casting gel interfaces with the glass plate), and substantial macrovoids in the central area. This observation can be attributed to a rapid demixing process occurring from the top side, a stagnation of solvent-antisolvent interaction in the middle, and a gradual demixing process at the bottom side. In Figure 7C, the upper portion of the PES/CNT/C5 membrane reveals a thin layer of finger-like structures, resembling those seen in PES/CNT/C3 (Figure 7B).

Pristine membranes produced with Cyrene (PES/C in Figure 7E) and NMP (PES/N in Figure 7F) show larger macrovoids compared to the same membranes produced with added SWCNTs (Figures 7A,D). However, the Cyrene-based membrane (Figure 7E) exhibits interconnected pores within these macrovoids, similar to all Cyrene and SWCNTs-based membranes (Figures 7A–C). However, in case of NMP-based membranes, these interconnected pores can only be seen when SWCNTs were employed (Figure 7D). Additionally, large macrovoids traverse the PES/CNT/N membrane (Figure 7D), while a modest presence of sponge-like structures is observed at the membrane’s centre. This observation suggests a rapid demixing process occurring at the edges of the membrane, with a notable deceleration in the central region. This slower demixing in the center is attributed to the higher viscosity of the casting solution caused by the presence of carbon nanotubes, which hinders the diffusion and separation process. The rapid demixing from the bottom side indicates a potential separation of the emerging membrane from the glass plate, allowing water to infiltrate the space between the plate as demixing initiates or a fast water leaching through the displaced side of the membrane (Supplementary Figure S9). On the upper surface, demixing occurs swiftly, leading to the precipitation of the polymer and the formation of voids due to water displacement. Comparable voids become apparent when the membrane is separated from the glass plate, allowing water to ingress into the space or release the membranes porosity (Supplementary Figure S9B). The uniform thickness of all membranes, set at only 150 μm, coupled with the introduction of the hydrophilic pore-forming additive, contributes to an accelerated demixing rate. PVP serves as an antisolvent agent during the demixing step, owing to its high solubility in water. Moreover, it is essential to note that the water-Cyrene demixing process deviates from conventional solvent/non-solvent demixing. When water is introduced to Cyrene, the resulting mixture transforms into a complex blend of Cyrene, water, and geminal diol (De Bruyn et al., 2019). Furthermore, the viscosity of this new mixture varies depending on the amount of water added to Cyrene, ranging from a low viscosity, similar to that of water, to a more viscous solution when only 25% water is present in Cyrene (Milescu et al., 2023). Throughout the demixing process, if the mixture contains more Cyrene than water at any given time, the viscosity of the solution increases, impacting pore formation. In this case, a higher viscosity would create macrovoids. Therefore, the formation of large macrovoids in PES/CNT/C3 and PES/CNT/C5 membranes may result from two contributing factors: 1) the hydrophilic nature of PVP, which facilitates faster demixing by interacting with water and local viscosity increase and 2) the viscosity of the water/Cyrene/diol combination during demixing, leading to the development of extensive zones at the contact interface.

**FIGURE 8:**
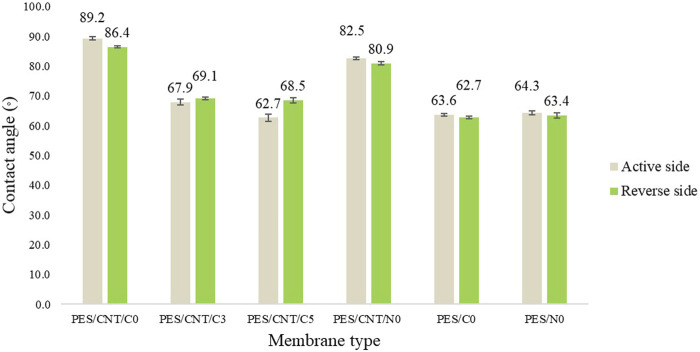
Average contact angles of Cyrene- and NMP-based membranes surfaces (active and reverse side).

The water contact angle of the flat sheet membranes was found to be influenced by the dispersion of carbon nanotubes within the polymer matrix (Figure 8; Supplementary Table S2).

As shown in Figure 8, PES membranes containing SWCNTs (PES/CNT/C0 and PES/CNT/N0) exhibited highest water contact angles (up to 89.2°) compared to those without SWCNTs (PES/C0 and PES/N0) with contact angle up to 64.3°. This increase can be attributed to the inherent hydrophobicity of the pristine nanotubes. The non-uniform dispersion of SWCNTs within the polymer matrix produced with NMP led to the formation of aggregates, which, in turn, caused a reduction in the contact angle of the PES/CNT/N0 membrane. These agglomerates likely altered the surface characteristics, making it more hydrophilic, as water could interact more readily with the exposed areas of the membrane. The inclusion of polyvinylpyrrolidone (PVP), a hydrophilic additive, in the casting solution further enhanced membrane hydrophilicity. Increasing PVP concentration significantly reduced the water contact angle, from an average of 68° for PES/CNT/C3 to 65° for PES/CNT/C5. The difference in contact angle between the active and reverse sides of the membranes can be attributed to the non-uniform dispersion of nanotubes during membrane fabrication. A higher concentration of nanotubes on the reverse side results in increased hydrophobicity on that surface.

**FIGURE 9:**
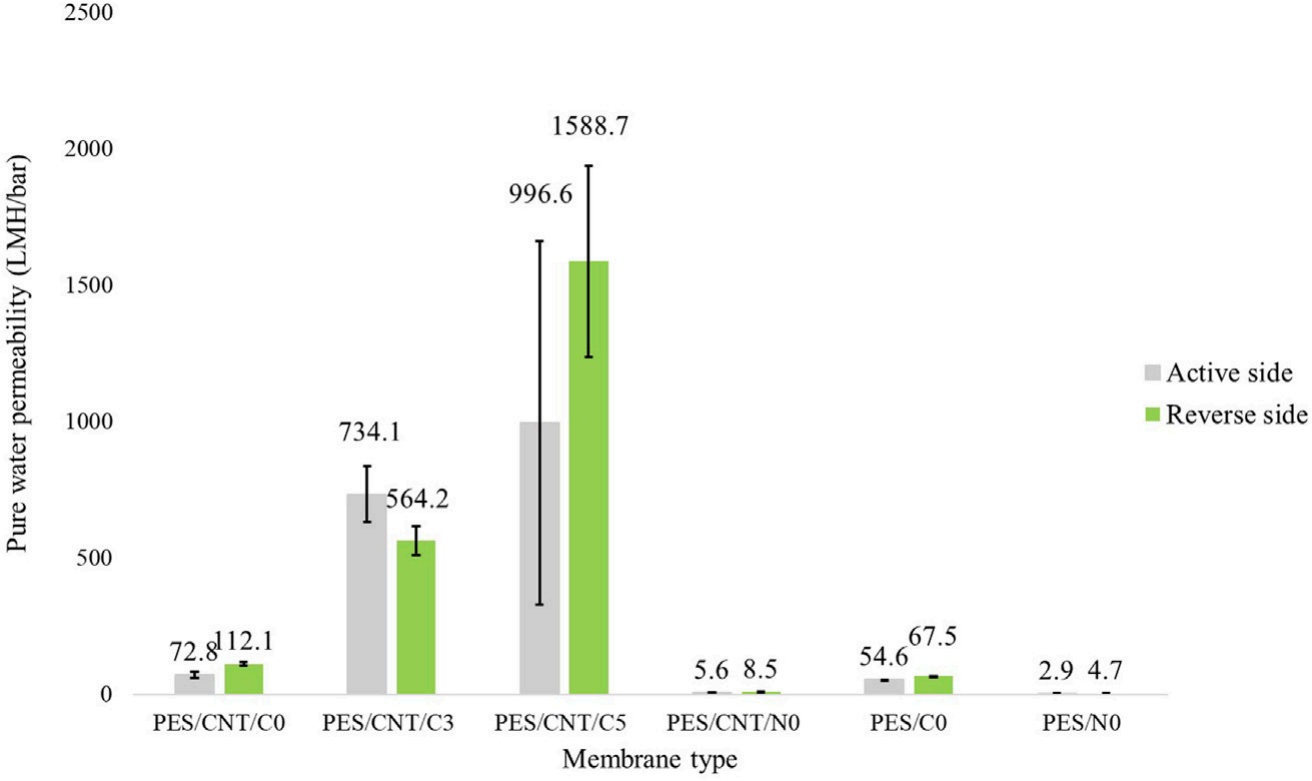
Pure water permeability of membranes cast in Cyrene/NMP containing carbon nanotubes. Two pristine membranes, PES/C0 and PES/N0, were tested as references. Both the active sides (shown in grey) and the reverse sides (shown in green) of the membranes were tested.

To explore the effects of membrane cross-section morphology on its permeability and the practical application of the prepared PES membranes with carbon nanotubes, their water permeation fluxes were investigated. The membranes were investigated using both sides, due to their different porosities: 1) active side (top) and reverse side (the side close to the glass plate). The average water permeability of the membranes can be seen in Figure 9.

The presence of macrovoids is considered a deviation from the desired membrane morphology, impacting filtration efficiency and potentially leading to superior filtration outcomes (Kosma and Beltsios, 2012). Conversely, the sponge structures present in the absence of PVP support a less effective filtration ability, which may be enhanced by increasing the applied pressure. Based on these criteria, the permeate flux seen in Figure 9 shows differences between the membranes depending on the solvent used, presence of SWCNTs and side of the membrane used. The passive side of the membrane generally exhibited higher permeability compared to the active side, aligning with the observed skin porosity in Supplementary Figure S10. All Cyrene-based membranes display pores on the passive side, which become larger when SWCNTs are added. In contrast, NMP-based membranes do not show any visible skin pores, when optical microscopy was employed. An exception was noted for PES/CNT/C3, where the active side displayed greater permeability than the passive side of the membrane. This could be attributed to either 1) a thicker sponge-like layer beneath the passive side, impeding filtration or 2) to a more hydrophilic active side (contact angle of 67.9°) compared to reverse side (69.1°). Additionally, finger layers in this membrane’s morphology feature large zones of end ends, adversely affecting the flux. All Cyrene-based membranes demonstrate significantly higher water permeance than their NMP-based counterparts. The PES/CNT/C0 membrane demonstrated a water permeability that was thirteen times higher than that of its NMP counterpart. This significant increase is attributed to the presence of large pores in the skin layer, as well as the large, interconnected macropores and the porous sponge layer of the Cyrene-based membrane. The addition of PVP resulted in even greater water permeability, attributed to the presence of large macrovoids. It is crucial to note, however, that these membranes are susceptible to collapse under high pressure, rendering them impractical for certain applications.

Adding carbon nanotubes to the casting solution increased the pore size on the membrane surface, enhancing water permeability and resulting in higher water permeability compared to pristine membranes. Cyrene-based membranes, which were operated at low pressures (less than 5 bar) as shown in Supplementary Table S3, are versatile and can be used in various filtration ranges: nanofiltration (10–100 LMH and 4–30 bar), ultrafiltration (50–200 LMH and 2–10 bar), and microfiltration (greater than 100 LMH and 0.1–2 bar). These membranes are suitable for applications involving the filtration of small molecules, proteins, viruses, and suspended solids in water treatment and food processing. In contrast, NMP-based membranes required higher pressures, up to 8 bar, to achieve efficient filtration.

### 3.4 Bacterial challenge of membranes and bukypapers

To assess if the membranes formed with and without SWCNT in both NMP and Cyrene displayed any difference in terms of bacterial activity, a simple bacterial challenge was devised whereby the membranes and buckypapers were exposed to a Gram-negative (*E. coli)* and a Gram-positive (*B.subtilis*) strain of bacteria. No active filtration was conducted, bacteria were taken up into the porous network of the membrane through adsorption with any free solution removed by mild centrifugation. Results are shown in Supplementary Figures S11, S12. Data shows that all membranes have sufficient porosity to uptake bacteria, but that the presence of SWNT did not show any significant difference to the controls in terms of bacterial colony count. The negative control of filter paper showed a higher colony count compared to the PES membranes, but no significant differences were observed between membranes cast in Cyrene or NMP, with or without the presence of SWCNT. Similarly, the presence of SWCNT supported on the surface of a nylon membrane did not show any significant difference to the negative control of just the nylon filter. To ensure the test was functioning, positive control of filter paper treated with a quaternary ammonium salt was also trialed. This sample showed no bacterial growth for both Gram-negative or Gram-positive bacteria. This shows that the presence of SWCNT doesn’t impart any antibacterial properties when using a simple challenge test.

### 3.5 Environmental impact and life cycle analysis

Cyrene, a bio-based solvent derived from cellulose, is generally considered safer and more environmentally friendly than traditional solvents like NMP (Milescu, 2021). Cyrene is non-mutagenic, biodegradable, and does not contain potentially harmful amide groups associated with reproductive toxicity, as observed with NMP. It also avoids the formation of harmful by-products during degradation, offering a safer profile compared to many petroleum-based solvents. Still, any residual Cyrene in materials, like filtration membranes and buckypapers, requires thorough investigation to ensure it does not leach into water or other fluids in concentrations that could compromise safety for human exposure. Carbon nanotubes, celebrated for their exceptional mechanical strength, electrical conductivity, and thermal stability, are remarkable nanomaterials with vast applications in fields like electronics, medicine, and environmental engineering. However, they are also linked to significant concerns, including toxicological risks, environmental persistence, workplace safety challenges, gaps in research and regulatory frameworks, and the ongoing need for mitigation strategies and safer alternatives. Integrating green solvents like Cyrene with CNT technology in filtration membranes and buckypapers, underpinned by robust green metrics and Life Cycle Assessment, offers a pathway to optimise performance while reducing environmental and health risks. Mass-based metrics like Process Mass Intensity (PMI) and E-factor are valuable tools for assessing the sustainability of production processes. PMI quantifies the total mass of inputs required to produce 1 kg of the desired product, while E-factor focuses on the mass of waste generated per kilogram of product (Martínez et al., 2022). These metrics are straightforward to calculate for optimised systems and provide clear benchmarks for resource efficiency and waste reduction. LCA considers environmental impacts across all stages of the product’s life, from raw material extraction to end-of-life disposal or recycling. That said, performing an LCA is most meaningful at the pilot or industrial scale, where processes and resource flows are sufficiently developed and stable to provide reliable data (Hong et al., 2023).

## 4 Conclusion

This study explores the transformative potential of nanotechnology, particularly emphasising the remarkable impact of carbon nanotubes and bio-based solvents. Carbon nanotubes have garnered significant attention due to their exceptional properties, including high mechanical strength, electrical conductivity, and thermal stability. Their crucial role in sustainable nanofluids, especially when integrated with eco-friendly solvents, holds promise for fostering enhanced sustainability in nanomaterials. The investigation into the dispersion of nanotubes in Cyrene and NMP solvents, along with the synthesis of surfactant-free buckypapers and filtration membranes, adds depth to our understanding of the practical applications of nanotechnology. Cyrene significantly enhances dispersion stability, enabling the production of denser buckypapers and high-performance PES membranes. This study demonstrates that Cyrene achieves superior dispersion and stability of SWCNTs compared to NMP, attaining nearly three times higher concentrations without requiring surfactants. Buckypapers fabricated using Cyrene exhibit enhanced structural characteristics, including tighter SWCNT packing and smoother surface morphology. These improvements translate into superior filtration performance, particularly in wastewater treatment and oil-water separation applications. This study underscores the importance of solvent choice in the development of high-performance nanotube-based membranes. Cyrene promotes a more uniform and compact distribution of SWCNTs, leading to potentially improved membrane properties, while NMP results in a more heterogeneous distribution. The variations in the nanofluid dispersion affected the porosity of membranes, their contact angle, and filtration efficiency. PES membranes made with Cyrene demonstrated superior water filtration capacity compared to those made with NMP, operating effectively at lower pressures across various filtration ranges (nanofiltration to microfiltration). In simple surface bacterial challenge testing, the presence of SWCNT showed no significant anti-bacterial activity. In a future study, these membranes will be employed in active filtering of media-containing bacteria. Under such conditions, the presence of a flow through the membranes may result in bacteria being driven onto the SWCNT and a resultant drop in bacterial count may be observed. Further studies investigating the effects of membrane-incorporated SWCNT on biofilm formation and microbial-mediated filter blinding may pave the way for future applications. These findings establish Cyrene as a promising green solvent for membrane and buckypaper fabrication, aligning with sustainability goals while maintaining or enhancing performance characteristics. Future research should focus on optimising processing conditions to minimise solvent residuals, exploring surface modifications for specific applications, and conducting comprehensive life cycle assessments at industrial scales. This work contributes significantly to the development of more sustainable membrane fabrication processes while highlighting the importance of careful consideration of safety and performance parameters in the transition to greener technologies.

## Data Availability

The original contributions presented in the study are included in the article/Supplementary Material, further inquiries can be directed to the corresponding authors.
