# Supplementary material for: Sustainable nanomaterials: the role of Cyrene in optimising carbon nanotubes dispersion and filtration efficiency
Source: Front Chem. 2024 Dec 19;12:1498279. doi: 10.3389/fchem.2024.1498279 (PMC11694148; doi:10.3389/fchem.2024.1498279)
Supplement: Supplementary file 1 [file DataSheet1.PDF]

Supplementary Material

Sustainable Nanomaterials: The Role of Cyrene in Optimising Carbon Nanotubes Dispersion and Filtration Efficiency

Roxana A. Milescu[1*], C. Rob McElroy[2], Edward J. Taylor[3], Peter Eaton[2,4], Paul M. Williams[5], Richard Phillips[6], Thomas J. Farmer[7], James H. Clark[1*]

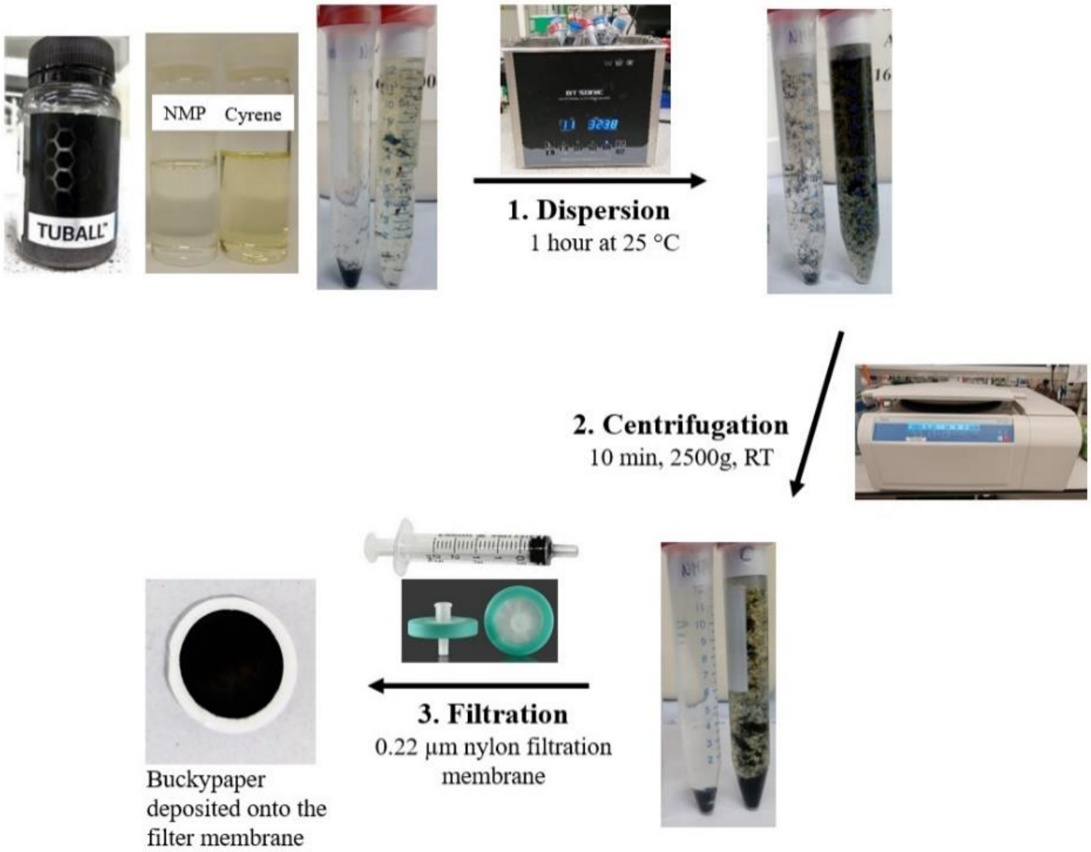

Supplementary Figure 1 Scheme of the dispersion of SWCNTs in neat solvents and the buckypaper formation.

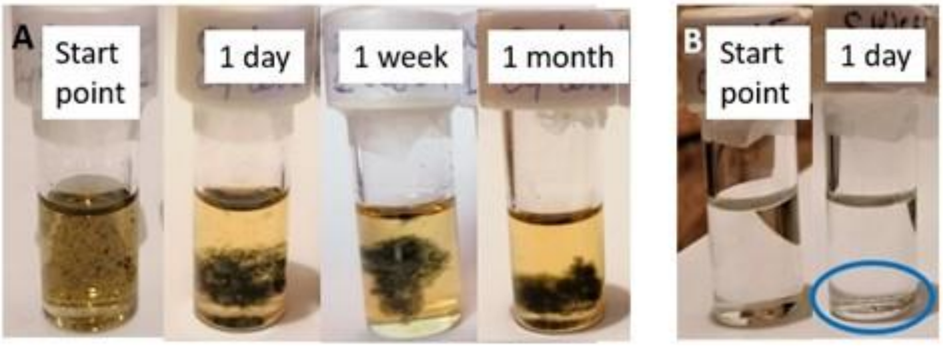

Supplementary Figure 2 Stability test of Cyrene (A) and NMP-based nanofluids (B) after dispersion and centrifugation. Only the supernatant is shown here.

Supplementary Table 1 Concentrations, average, and standard deviation of single-walled carbon nanotubes (SWCNTs) dispersed in neat Cyrene and NMP.

**Solvent**	**Test 1** (mg mL^{-1})	**Test 2** (mg mL^{-1})	**Test 3** (mg mL^{-1})	**Test 4** (mg mL^{-1})	**Average** (mg mL^{-1})	**Standard deviation**
Cyrene	0.067	0.02	0.054	0.011	0.038	0.027
NMP	0.012	0.014	0.012		0.013	0.001

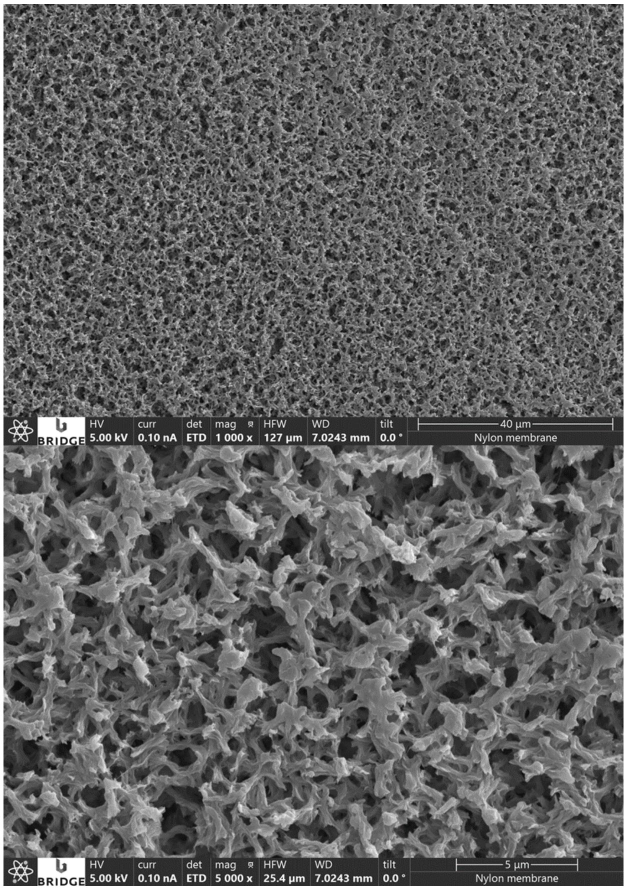

Supplementary Figure 3 Additional SEM images of the nylon membrane used for buckypapers

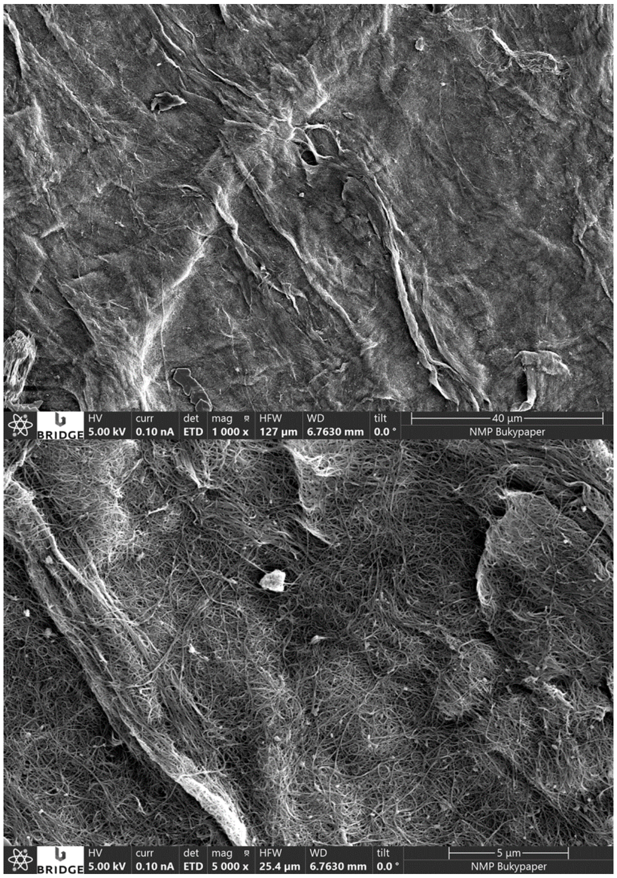

Supplementary Figure 4 Additional SEM images of NMP Buckypaper

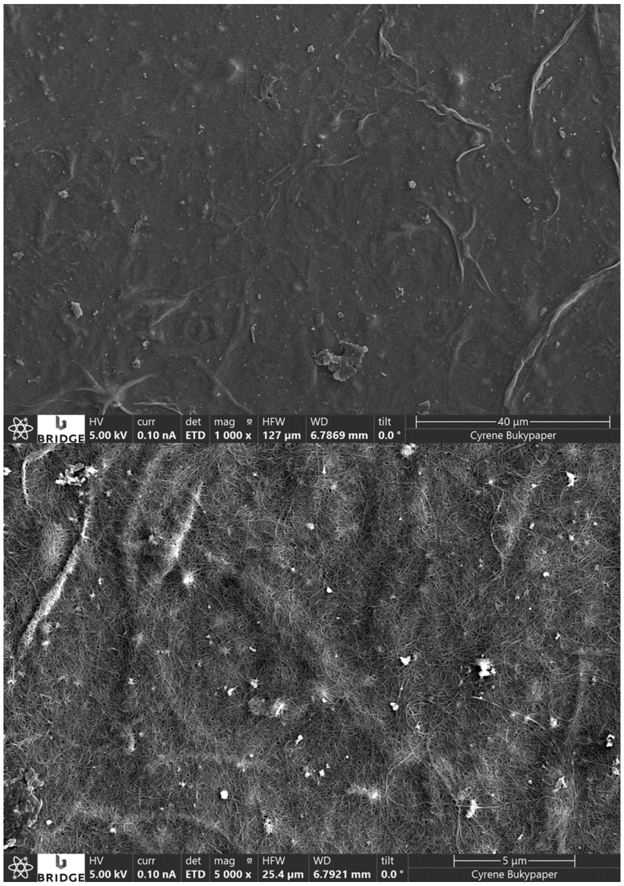

Supplementary Figure 5 Additional SEM images of Cyrene Buckypaper

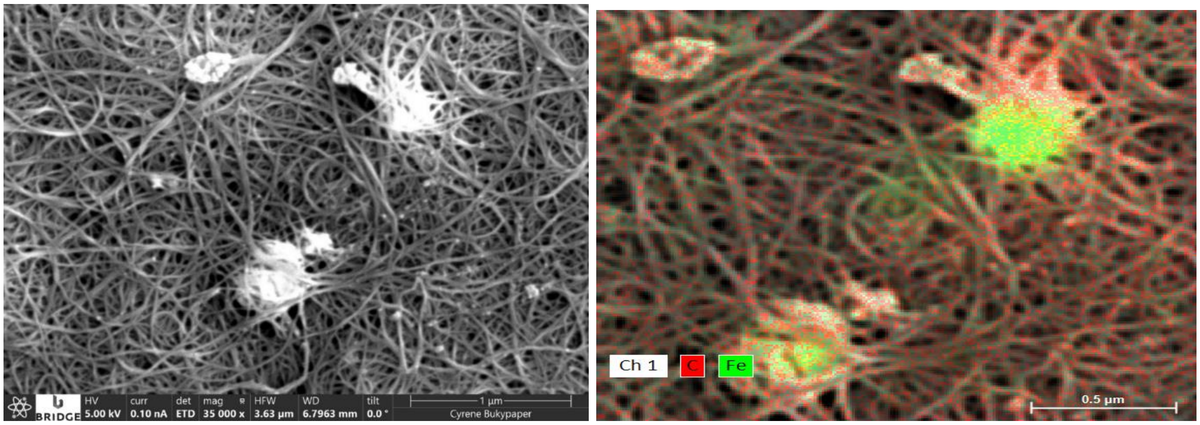

Supplementary Figure 6 SEM (left) and EDX (right) images of impurities on Cyrene buckypaper

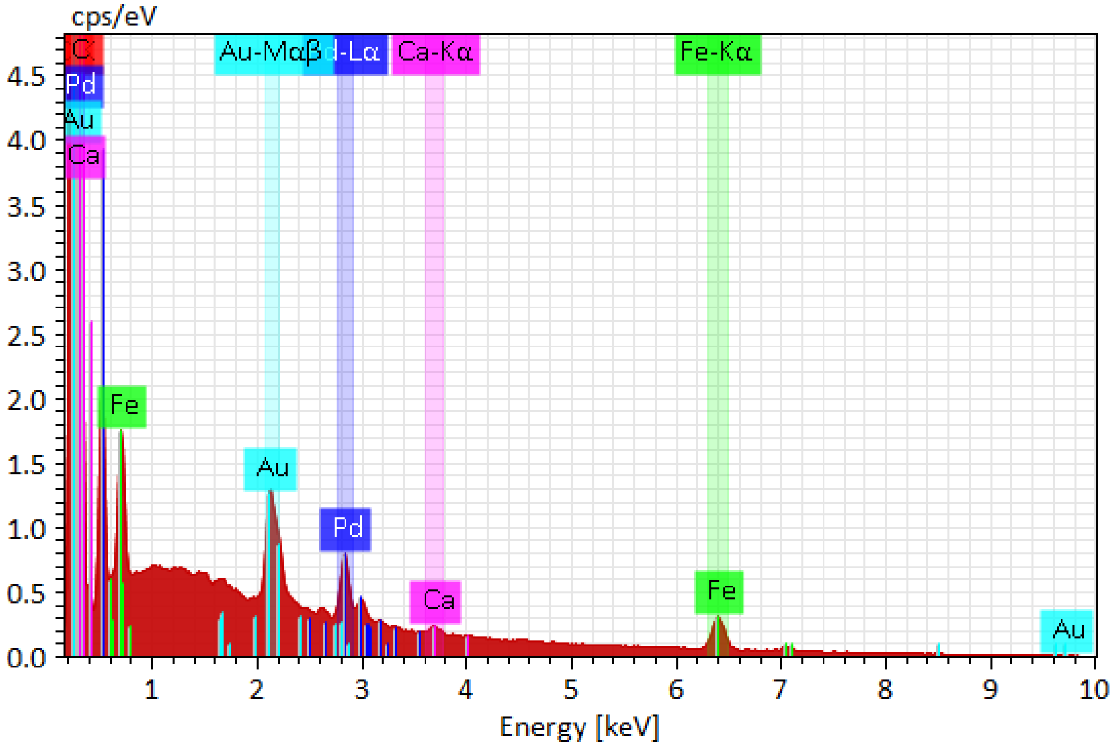

Supplementary Figure 7 EDX spectrum for the area containing impurities on Cyrene buckypaper

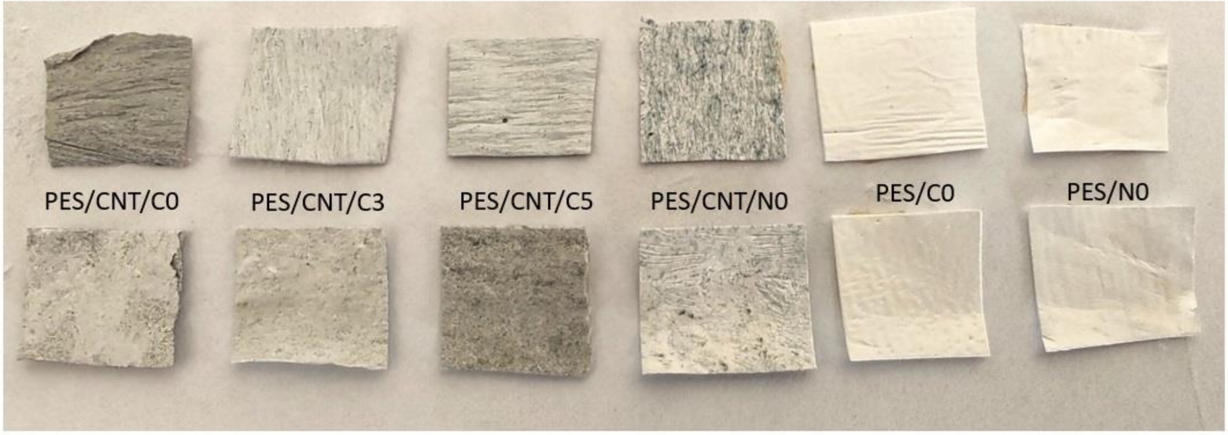

Supplementary Figure 8 Flat sheet membranes (active side - top and reverse side - bottom) of PES produced with Cyrene or NMP, with 3 or 5% PVP or NMP. Pristine PES membranes, without carbon nanotubes can also be seen here.

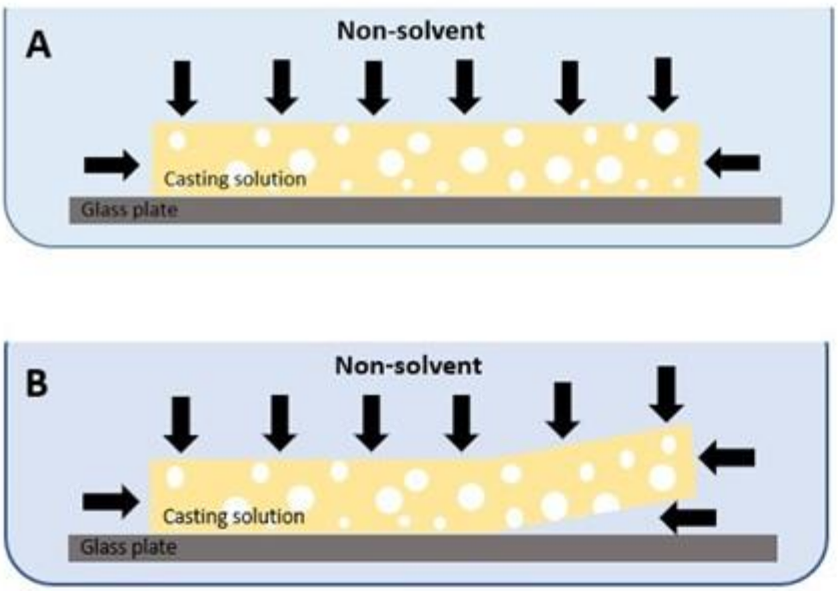

Supplementary Figure 9 Typical progression of the demixing process (A), and initiation of demixing when the antisolvent begins permeating the space between the casting plate and the membrane (B).

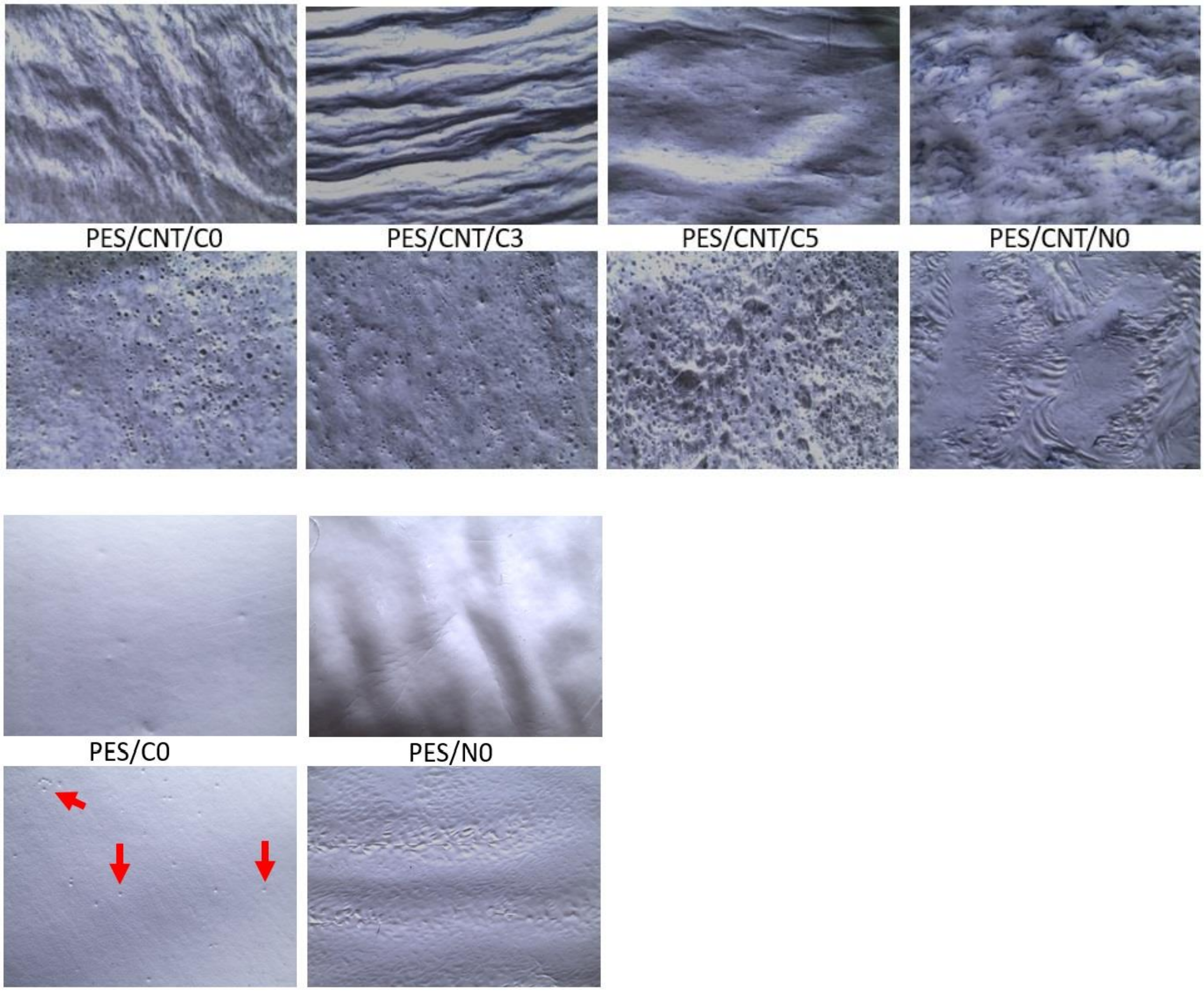

Supplementary Figure 10 Stereo (optical) microscope images of the active side (top) and reverse side (bottom) of PES membranes produced with Cyrene or NMP, with 3 or 5% PVP or NMP. Pristine PES membranes, without carbon nanotubes have been also produced.

Supplementary Table 2 Contact angle of polymeric PES membranes cast in Cyrene and NMP, both active and reverse sides. their average and standard deviations (STDEV).

Sample	Active side 1	Active side 2	Active side 3	Average	STDEV	Reverse side 1	Reverse side 2	Reverse side 3	Average	STDEV
PES/CNT/C0	89.7	89.4	88.6	89.2	0.6	86.2	86.8	86.2	86.4	0.3
PES/CNT/C3	68.9	67.8	67	67.9	1.0	69.3	68.7	69.4	69.1	0.4
PES/CNT/C5	61.3	63.4	63.3	62.7	1.2	67.5	68.8	69.1	68.5	0.9
PES/CNT/N0	82.6	82.1	82.9	82.5	0.4	81.2	80.2	81.3	80.9	0.6
PES/C0	64	63.8	63.1	63.6	0.5	62.2	63.1	62.9	62.7	0.5
PES/N0	64.4	64.9	63.6	64.3	0.7	62.3	63.8	64.1	63.4	1.0

Supplementary Table 3 Pure water permeability of membranes cast in Cyrene and NMP. Both active and reverse sides of the membranes have been tested.

**Pressure (Bar)**	**PES/CNT/C0 Active (LMH/bar)**	**Pressure (Bar)**	**PES/CNT/C3 Active (LMH/bar)**	**Pressure (Bar)**	**PES/CNT/C5 Active (LMH/bar)**	**Pressure (Bar)**	**PES/CNT/N0 Active (LMH/bar)**
1.004	47.9	0.496	937.2	0.211	2083.4	2.017	8.7
2.001	80.1	0.996	793.5	0.506	1534.9	3.997	8.5
2.978	77.4	1.489	687.2	0.763	1170.5	5.995	7.4
3.998	76.8	1.99	639.7	0.985	975	7.959	5.8
5.004	77.8	1.49	681.4	0.747	661.3	5.967	4.9
3.995	85.5	0.99	685.6	0.519	350.9	3.999	2.4
2.986	83.2	0.5	713.9	0.218	200	1.996	1.8
1.996	69.7						
1.003	56.8						
**Average**	72.8		734.1		996.6		5.6
**STDEV**	12.6		101.2		666.5		2.8
**Pressure (Bar)**	**PES/CNT/C0 Reverse (LMH/bar)**	**Pressure (Bar)**	**PES/CNT/C3 Reverse (LMH/bar)**	**Pressure (Bar)**	**PES/CNT/C5 Reverse (LMH/bar)**	**Pressure (Bar)**	**PES/CNT/N0 Reverse (LMH/bar)**
0.997	124.1	0.504	660.8	0.227	1759.8	1.994	9.7
1.986	117.6	1.019	588.5	0.508	1579.2	3.967	9.8
3.004	114.3	1.504	532.8	0.748	1337.5	5.985	9.2
3.998	116.3	1.988	497.2	0.992	1270.6	8	10
5.006	101.1	1.423	548.4	0.756	1402.3	5.971	8.6
3.993	109.3	0.993	541	0.494	1480	4.002	6.7
2.987	108.1	0.487	580.8	0.198	2291.4	2.013	5.3
1.994	111.4						
0.997	106.9						
**Average**	112.1		564.2		1588.7		8.5

**STDEV**	6.8		52.4		349.8		1.8
**Pressure (Bar)**	**PES/C0 Active (LMH/bar)**	**Pressure (Bar)**	**PES/N0 Active (LMH/bar)**	**Pressure (Bar)**	**PES/C0 Reverse (LMH/bar)**	**Pressure (Bar)**	**PES/N0 Reverse (LMH/bar)**
1.264	52.8	2.474	3.5	1.945	68.4	2.647	3.2
2.037	53.5	4.272	3.1	2.46	65.6	4.176	4.1
3.283	55.3	6.146	2.7	3.741	68.7	5.297	5.6
4.284	56.1	7.947	3.1	4.739	67.3	6.095	4.8
2.844	54.3	5.733	2.4	2.495	66.7	4.096	4.1
2.01	55.2	3.172	3.7	2.038	67.9	3.026	5.4
1.537	55.3	1.749	2.1	1.834	68.1	1.942	5.9
**Average**	54.6		2.9		67.5		4.7
**STDEV**	1.2		0.6		1.11		.0

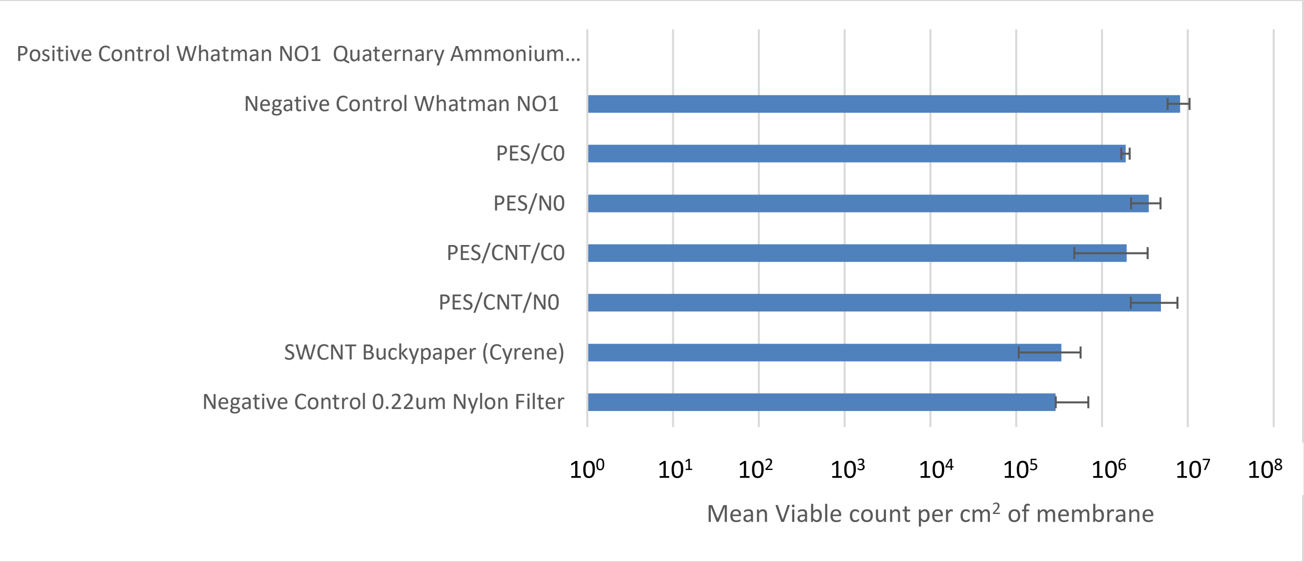

Supplementary Figure 11 Survival of *E.coli* on synthetic membranes. Membranes were challenged with a 7.0 x 10^6 to 3.0 x 10^7 culture of *E.coli* XL10-Gold® (Agilent) in LB media followed by a 6 hr incubation at 37 °C. The experiment was conducted in triplicate and the mean viable count per cm^2 was calculated along with the standard error. Whatman NO1 filter paper was used as a negative control, and Whatman NO1 filter paper treated with a quaternary ammonium disinfectant, was used as a positive control.

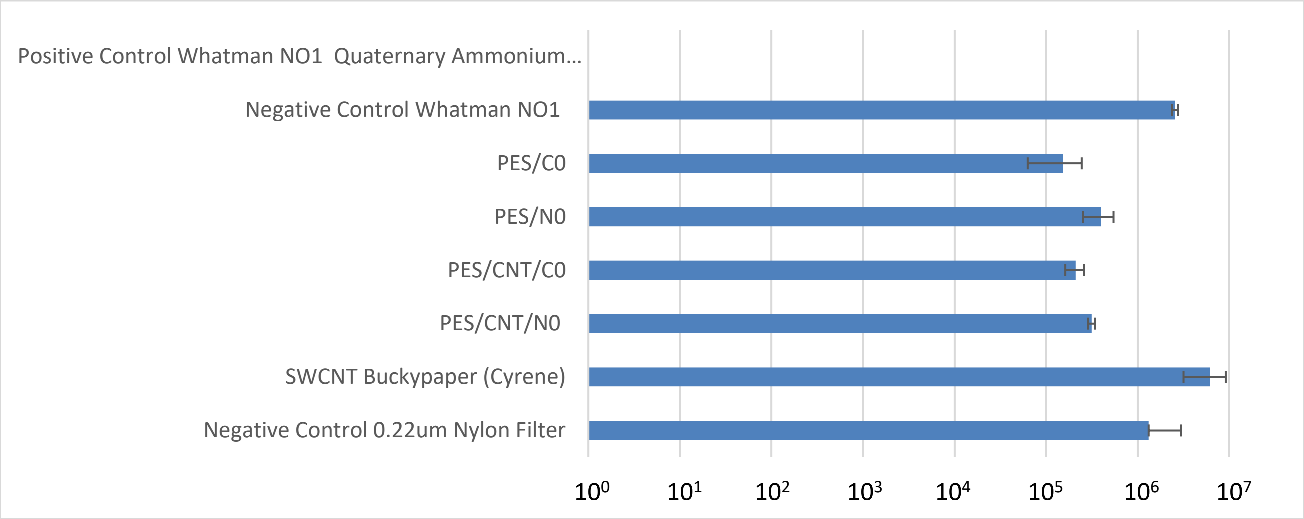

Supplementary Figure 12 Survival of *B.subtilis* on synthetic membranes. Membranes were challenged with a 1.5 x 10^6 to 3.7 x 10^6 culture of *B. subtilis* 168 in LB media followed by a 6 hr incubation at 37 °C. The experiment was conducted in triplicate and the mean viable count per cm^2 was calculated along with the standard error. Whatman NO1 filter paper was used as a negative control, and Whatman NO1 filter paper treated with a quaternary ammonium disinfectant, was used as a positive control
